# PDZK1‐ULK1 Axis Triggers Lipophagy to Inhibit Tumor Progression and Sunitinib Resistance in Clear Cell Renal Cell Carcinoma

**DOI:** 10.1002/advs.202511606

**Published:** 2026-02-16

**Authors:** Xuan Qi, Yu Guo, Yumeng Yang, Haibo Wang, Xiaomei Yang, Ran Song, Qiong Qin, Yan Zhang, Meihan Hu, Haixing Zhou, Duiping Feng, Junqi He

**Affiliations:** ^1^ Department of Biochemistry and Molecular Biology Beijing Key Laboratory for Tumor Invasion and Metastasis Capital Medical University Beijing China; ^2^ Laboratory for Clinical Medicine Capital Medical University Beijing China; ^3^ Department of Interventional Radiology First Hospital of Shanxi Medical University Taiyuan China

**Keywords:** lipophagy, PDZK1, sunitinib resistance, therapeutic targeting, ULK1

## Abstract

Clear cell renal cell carcinoma (ccRCC) is characterized by aberrant lipid droplet (LD) accumulation, which promotes tumor progression and sunitinib resistance. However, the underlying molecular mechanisms remain incompletely understood. This study shows that reduced PDZK1 expression correlates with LD accumulation and poor prognosis in ccRCC patients. Single‐cell RNA sequencing indicates that downregulated PDZK1 expression associates with impaired LD degradation in ccRCC cells. Functional studies demonstrate that PDZK1 inhibits LD accumulation by upregulating ULK1 expression and activating lipophagy, indicating the PDZK1‐ULK1 axis as a therapeutic target to enhance sunitinib efficacy. Mechanistically, CUT&Tag analysis reveals that LEF1 directly binds to the ULK1 promoter. PDZK1 interacts with LEF1 via its C‐terminus, sequestering LEF1 in the cytoplasm, thereby enhancing ULK1 transcription and autophagy activity. Pharmacological ULK1 activation with LYN‐1604 restores sunitinib sensitivity in PDZK1‐knockdown cells and synergizes with sunitinib in xenograft models, reducing tumor growth and LD accumulation. Clinical data demonstrate a strong correlation between ULK1 expression levels in tumor tissues and sunitinib response (AUC = 0.9063), suggesting its potential as a predictive biomarker. Collectively, the PDZK1‐ULK1 axis regulates LD homeostasis in ccRCC. Targeting this axis via ULK1 activation represents a novel strategy to overcome sunitinib resistance, with ULK1 as a potential biomarker for sunitinib efficacy.

## Graphical abstract

This study reveals that PDZK1 loss impairs ULK1‐mediated lipophagy, driving lipid droplet accumulation and sunitinib resistance in clear cell renal cell carcinoma. PDZK1 inhibits LEF1 nuclear translocation, enhancing ULK1 transcription. Pharmacological ULK1 activation resensitizes resistant tumors to sunitinib. ULK1 expression strongly correlates with clinical response, establishing it as a therapeutic target and predictive biomarker.

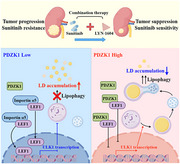

## 1 Introduction

Renal cell carcinoma (RCC) is a prevalent malignant tumor of the urinary system, with an estimated 434 840 new cases globally in 2022 [1]. Among its histological subtypes, clear cell renal cell carcinoma (ccRCC) is the most common, accounting for 75%–80% of all RCC cases. A pathological feature of ccRCC is the intracellular accumulation of lipid droplets (LDs), a phenomenon that significantly contributes to tumor progression and resistance to therapy [2, 3, 4].

Traditionally regarded as passive lipid storage compartments, LDs are now recognized as dynamic organelles involved in lipid metabolism. Through interactions with various organelles, LDs regulate oxidative stress, reduce endoplasmic reticulum (ER) stress, and maintain metabolic homeostasis [5]. In cancer cells, these functions are enhanced to meet the heightened metabolic demands of rapidly proliferating tumors, with LDs preserving ER integrity, scavenging reactive oxygen species (ROS), and supporting tumor cell survival and growth, emphasizing their importance in cancer pathophysiology [5, 6].

Structurally, LDs are enclosed by a phospholipid monolayer interspersed with surface proteins crucial for their metabolic and structural functions [5]. The lipid core contains neutral lipids, such as cholesteryl esters (CEs) and triglycerides (TGs), which serve as reservoirs for energy and membrane synthesis. In ccRCC, abnormal LD accumulation is integral to the metabolic reprogramming that supports tumor growth. Cholesterol‐enriched LDs promote ccRCC progression by supporting tumor cell proliferation and survival [7]. Moreover, LD storage regulated by the HIF‐2α pathway sustains ER homeostasis and tumor cell viability in ccRCC.

Aberrant lipid accumulation in ccRCC arises from a combination of increased lipid biosynthesis and impaired lipid catabolism. Lipophagy, a selective form of autophagy, is crucial for the degradation of LDs [6]. This process involves the sequestration of LDs into autophagosomes, which subsequently fuse with lysosomes for enzymatic degradation, releasing free fatty acids (FFAs) into the cytoplasm [8, 9]. However, in ccRCC, lipophagy is often impaired, leading to pathological LD accumulation that promotes tumor progression and therapy resistance [10].

Given the critical role of LDs in ccRCC biology, targeting lipid droplet homeostasis offers a potential therapeutic strategy. Recent studies have shown that targeting key enzymes involved in LD formation and turnover might be effective. For instance, inhibition of diacylglycerol acyltransferase 1 (DGAT1), an enzyme essential for LD biogenesis, has demonstrated therapeutic efficacy in preclinical glioblastoma models by disrupting LD formation, increasing ROS, and inducing apoptosis [11]. Similarly, pharmacological inhibition of stearoyl‐CoA desaturase 1 (SCD1), which facilitates LD formation to alleviate ER stress, sensitizes resistant gastric cancer cells to chemotherapy [12]. These findings highlight the therapeutic promise of disrupting LD homeostasis to impair the metabolic advantage of tumors. While these findings suggest that inhibiting lipid deposition and enhancing lipid utilization pathways, such as lipophagy, might be promising avenues for ccRCC treatment, the mechanisms underlying LD degradation in ccRCC have yet to be fully elucidated. Notably, ULK1 (Unc‐51 like autophagy activating kinase 1), a key regulator of autophagy and lipophagy [13, 14], plays an important role in LD degradation, yet its role in ccRCC lipid metabolism remains poorly understood.

In our study, we identified PDZK1 as a novel gene related to LD degradation and patient prognosis in ccRCC. Overexpression of PDZK1 activates autophagy‐dependent LD degradation by upregulating ULK1, thereby suppressing tumor growth and reversing resistance to sunitinib in vitro and in vivo. Mechanistically, PDZK1 interacts with LEF1, a transcriptional repressor of ULK1, preventing its nuclear translocation and relieving its transcriptional repression of ULK1. Overall, our findings establish the PDZK1‐ULK1 axis as a novel regulator in ccRCC biology. Targeting this axis by activating ULK1 represents a novel strategy to overcome sunitinib resistance. Moreover, ULK1 expression could serve as a potential biomarker for patient stratification, providing a pathway to optimize sunitinib‐based therapies.

## 2 Results

### 2.1 PDZK1 Suppresses Lipid Droplet Accumulation in ccRCC

Clear cell renal cell carcinoma (ccRCC) is characterized by excessive lipid droplet (LD) accumulation, which correlates with poor prognosis [15, 16]. To identify key modulators of LD degradation, we assembled a gene panel from KEGG encompassing the entire lipid catabolic pathway, from lipid droplet–specific degradation to subsequent fatty acid oxidation.

**FIGURE 1:**
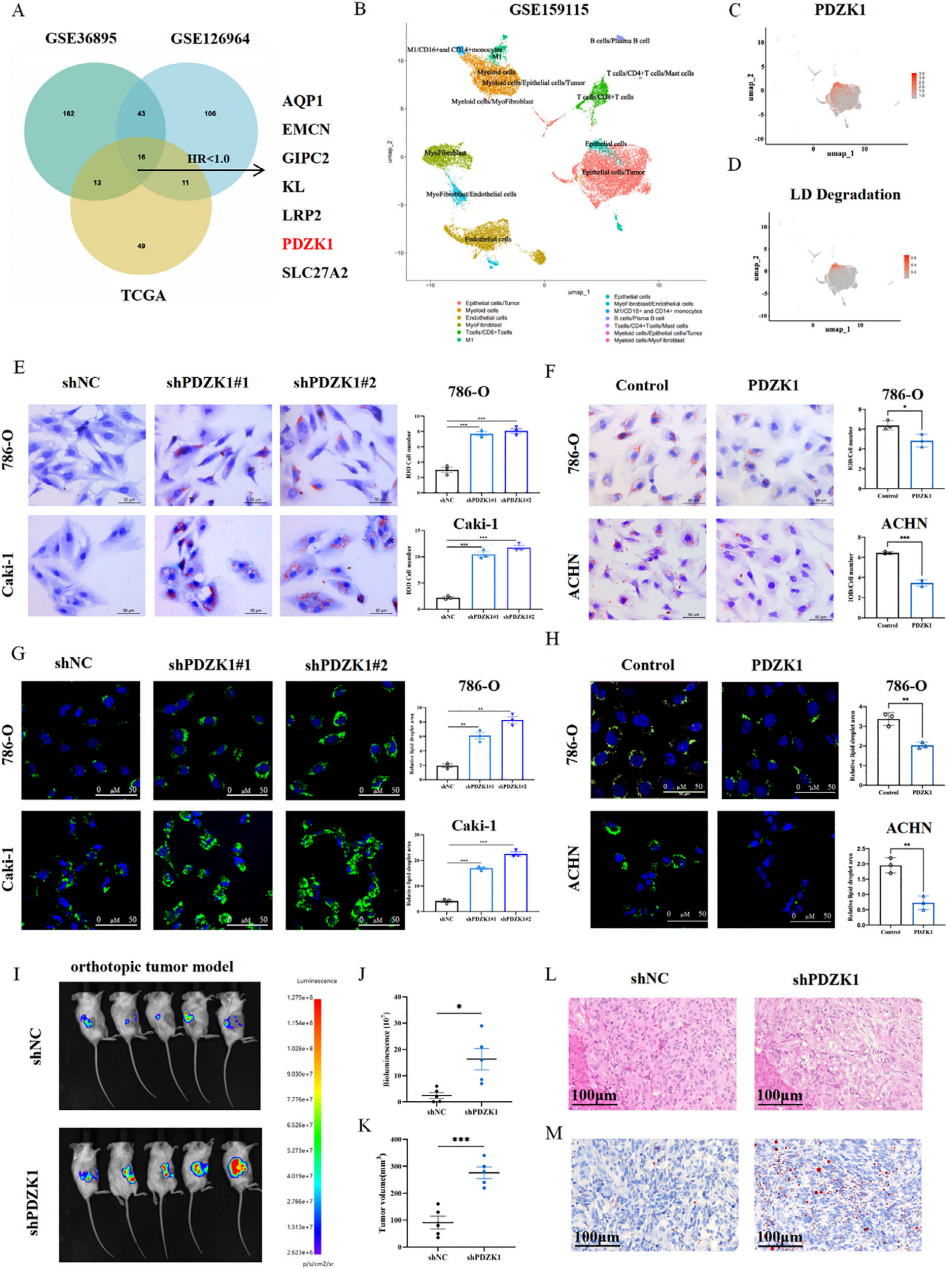
PDZK1 inhibits lipid accumulation in ccRCC. (A) Identification of differentially expressed genes associated with LD degradation and prognosis. The Venny diagram showed the 16 overlapped DEGs. Among these, seven genes with hazard ratios (HR) < 1 were selected for further evaluation of their association with overall survival in ccRCC patients. (B) UMAP visualization of 141 950 single cells profiled by scRNA‐seq from the GSE159115 dataset, colored by 13 cell clusters. (C,D) UMAP plot showing the distribution and expression of PDZK1 (C) and the GSVA score of LD degradation (D) in epithelial cell subpopulation, calculated using AddModuleScore. (E,F) Oil Red O staining of 786‐O, Caki‐1, and ACHN cells treated with 200 µm oleic acid (OA) and transfected with vector, (E) shPDZK1, or (F) PDZK1 overexpression. Scale bars, 50 µm. Data are presented as mean ±SD (3 replicates). (G,H) BODIPY staining in 786‐O, Caki‐1, and ACHN cells treated with 200 µm OA and transfected with vector, (G) shPDZK1 or (H) PDZK1 overexpression. Scale bars, 100 µm. Data are presented as mean ±SD (3 replicates). (I–K) PDZK1 knockdown reduced tumor burden in vivo. The representative bioluminescence images of orthotopic xenografts (I). Quantitative analysis of bioluminescence signals (J). Tumor weights were shown in K. Data are presented as mean ± SD (5 replicates). (L) Representative H&E staining of tumors from PDZK1 knockdown and control groups. (M) Oil Red O staining of orthotopic kidney tumors from PDZK1 knockdown and control groups. Scale bar: 100 µm. In all statistical plots, data are shown as mean ± SD, one‐way ANOVA (Figure 1E,G) and Two‐tailed unpaired Student's t test (Figure 1F,H,J,K) were used to determine statistical significance (^*^ *p* < 0.05, ^**^ *p *< 0.01, ^***^ *p *< 0.001).

Using Gene Set Variation Analysis (GSVA), we then calculated an LD degradation score for each tumor sample. To identify differentially expressed genes (DEGs) linked to LD turnover, tumors in two independent GEO cohorts (GSE36985 and GSE126964) were stratified into high‐ and low‐scoring groups based on these GSVA scores. Finally, by overlaying these DEGs with survival‐associated genes from the TCGA ccRCC cohort, we identified seven candidates whose high expression is associated with both enhanced LD degradation and improved overall survival (Figure 1A; Table S1).

Given the potential clinical relevance of the seven candidate genes, we further investigated their prognostic relevance. Survival analysis indicated that only high expression of PDZK1 and SLC27A2 was significantly associated with improved patient prognosis. Multivariable Cox regression model demonstrated that both PDZK1 and SLC27A2 remain independent favorable prognostic factors (Figure S1B). We further performed cell viability assays that showed PDZK1 knockdown induced a greater increase in cell proliferation than SLC27A2 knockdown in 786‐O and Caki‐1 cells (Figure S1C,D). Together, these results suggest that PDZK1 is the strongest candidate gene associated with LD degradation and favorable clinical outcomes in ccRCC.

Considering the heterogeneity of the tumor microenvironment, which influences metabolic profiles and gene expression across cell subpopulations, we utilized single‐cell RNA sequencing (scRNA‐seq) data (GSE159115) to explore the cell‐type‐specific relationship between PDZK1 and LD degradation (Figure 1B–D). Notably, PDZK1 expression was largely restricted to epithelial cells (Figure 1C,D), and cells with high PDZK1 expression showed higher LD degradation scores, suggesting a positive association between PDZK1 expression and LD degradation in epithelial cells (Figure S1D). These findings were further supported by Gene Set Enrichment Analysis (GSEA) using bulk‐RNA datasets (TCGA ccRCC and GSE53757), which showed that tumors with low PDZK1 expression were enriched for lipid accumulation signatures (Figure S1E).

To assess the effect of PDZK1 on LD accumulation in ccRCC cells, we performed both loss‐ and gain‐of‐function studies in vitro. Knockdown of PDZK1 increased LD deposition, as shown by Oil Red O staining (Figure 1E). In contrast, overexpression of PDZK1 reduced LD levels (Figure 1F). BODIPY staining further confirmed enhanced LD accumulation upon PDZK1 depletion and reduced LD levels when PDZK1 was overexpressed (Figure 1G,H). Additionally, quantitative measurement of cellular triglycerides, the main component of lipid droplets, revealed elevated levels in PDZK1‐knockdown cells and decreased levels in PDZK1‐overexpressing cells (Figure S1F–I).

To investigate the role of PDZK1 in vivo, we established an orthotopic kidney tumor model using PDZK1 knockdown and control 786‐O cells. PDZK1 knockdown significantly inhibited tumor growth compared to the control group (Figure 1I–K). Histopathological analysis confirmed that PDZK1 knockdown tumors retained the typical clear cell morphology of ccRCC (Figure 1L). Importantly, Oil Red O staining showed increased LD accumulation in PDZK1 knockdown tumors compared with controls (Figure 1M; Figure S1J). Taken together, these results demonstrate that PDZK1 attenuates LD accumulation in ccRCC by promoting LD degradation.

### 2.2 PDZK1 Activates Autophagy to Facilitate LD Degradation in ccRCC Cells

**FIGURE 2:**
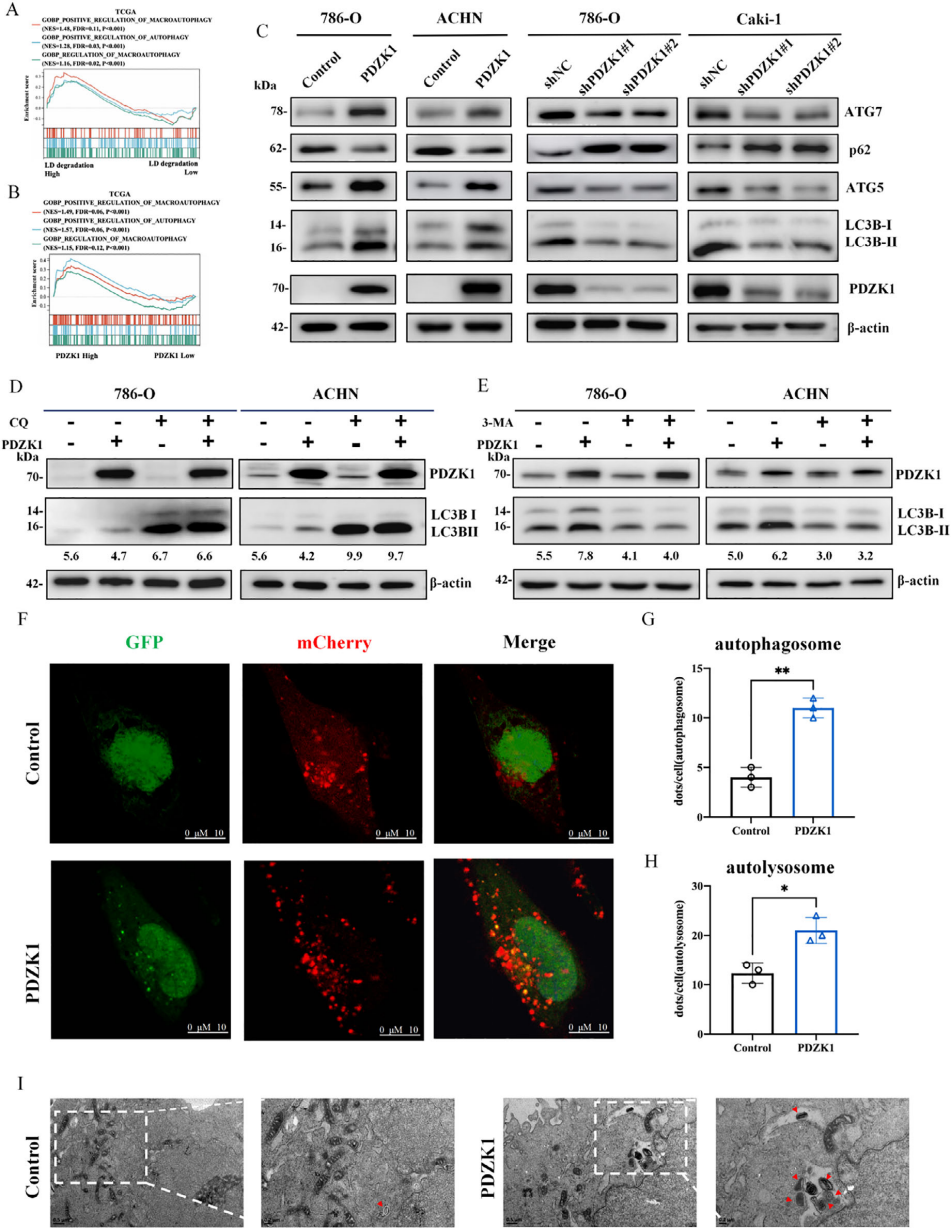
PDZK1 activates autophagy in ccRCC cells. (A,B) Enrichment plots of GSEA showing significant enrichment of autophagy‐related pathways in ccRCC specimens with high LD degradation score (A) or high PDZK1 expression (B) from the TCGA dataset. We calculated the LD degradation score for each tumor sample using GSVA analysis. (C) Western blot analysis illustrating the impact of PDZK1 overexpression or knockdown on autophagic flux in 786‐O, Caki‐1, and ACHN cell lines. (D) The confirmation of autophagy activation using chloroquine (CQ) in control or PDZK1 knockdown 786‐O and 769‐P cells, both with and without CQ treatment (50 nm, 24 h). (E) The confirmation of autophagy activation inhibition using 3‐MA in control or PDZK1 overexpressing 786‐O and ACHN cells, both with and without 3‐MA treatment (2 mm, 24 h). (F–H) Representative confocal microscopy images of 786‐O cells stably expressing GFP‐mCherry‐LC3 after EBSS treatment (F). Co‐localization of GFP and mCherry (yellow) indicate autophagosomes (G); red puncta indicate autolysosomes (H). Data were shown as mean ± SD. Scale bar, 10 µm. (I) Electron microscopy images showing autophagic structures in 786‐O cells with PDZK1 overexpression compared to control cells. Scale bar, 500 nm. In all statistical plots, data are shown as mean ± SD, Two‐tailed unpaired Student's t test (Figure 2G,H) was used to determine statistical significance (^*^ *p* < 0.05, ^**^ *p *< 0.01).

Lipid droplet (LD) degradation in cells depends on two primary mechanisms: lipophagy, where autophagy transports LDs to lysosomes for hydrolysis, and neutral lipolysis, mediated by cytosolic lipases [17]. To determine which pathway predominates in ccRCC cells, we performed GSEA on LD‐degradation GSVA scores from the TCGA database. Our findings revealed that patients with high LD‐degradation scores showed significant enrichment of autophagy pathways (Figure 2A), while lipolysis pathway remained unaltered (Figure S2A). These results suggest that lipophagy plays a critical role in LD degradation in ccRCC cells. Additionally, patients with high PDZK1 expression also showed enrichment of autophagy pathways (Figure 2B; Figure S2B). We therefore proposed that PDZK1 might facilitate LD degradation by promoting autophagy in ccRCC cells.

To test this hypothesis, we assessed the effect of PDZK1 expression on autophagy activity in ccRCC cells. Overexpression of PDZK1 markedly elevated the LC3B‐II/LC3B‐I ratio and upregulated ATG5 and ATG7 expression, while decreasing p62 levels, indicating enhanced autophagy flux. In contrast, PDZK1 knockdown reduced ATG5 and ATG7 expression as well as the LC3B‐II/LC3B‐I ratio, and increased p62 levels (Figure 2C; Figure S2C). To further explore the role of PDZK1 in autophagy regulation, we employed the lysosomal degradation inhibitor chloroquine (CQ) and observed that LC3B‐II levels remained elevated after treatment, suggesting that PDZK1 promotes autophagosome formation. Additionally, treatment with the autophagosome inhibitor 3‐methyladenine (3‐MA) also abolished the increased ratio in PDZK1‐overexpressing cells, indicating that PDZK1 acts at the initiation stage of autophagy rather than by preventing lysosomal degradation (Figure 2D,E).

To verify this result, we employed the mCherry‐GFP‐LC3 reporter and observed that PDZK1 overexpression significantly increase in both yellow puncta (autophagosomes) and red‐only puncta (autolysosomes) compared to controls. These findings suggest that PDZK1 promotes both autophagosome biogenesis and downstream autolysosomal maturation (Figure 2F–H). Transmission electron microscopy further confirmed a marked increase in autophagosome vesicles in PDZK1‐overexpressing cells (Figure 2I).

Together, these data indicate that PDZK1 activates autophagy in ccRCC cells, potentially facilitating lipophagy and thereby promoting efficient LD degradation.

### 2.3 PDZK1 Enhances Autophagy‐Mediated Lipid Droplet Clearance in ccRCC Cells

**FIGURE 3:**
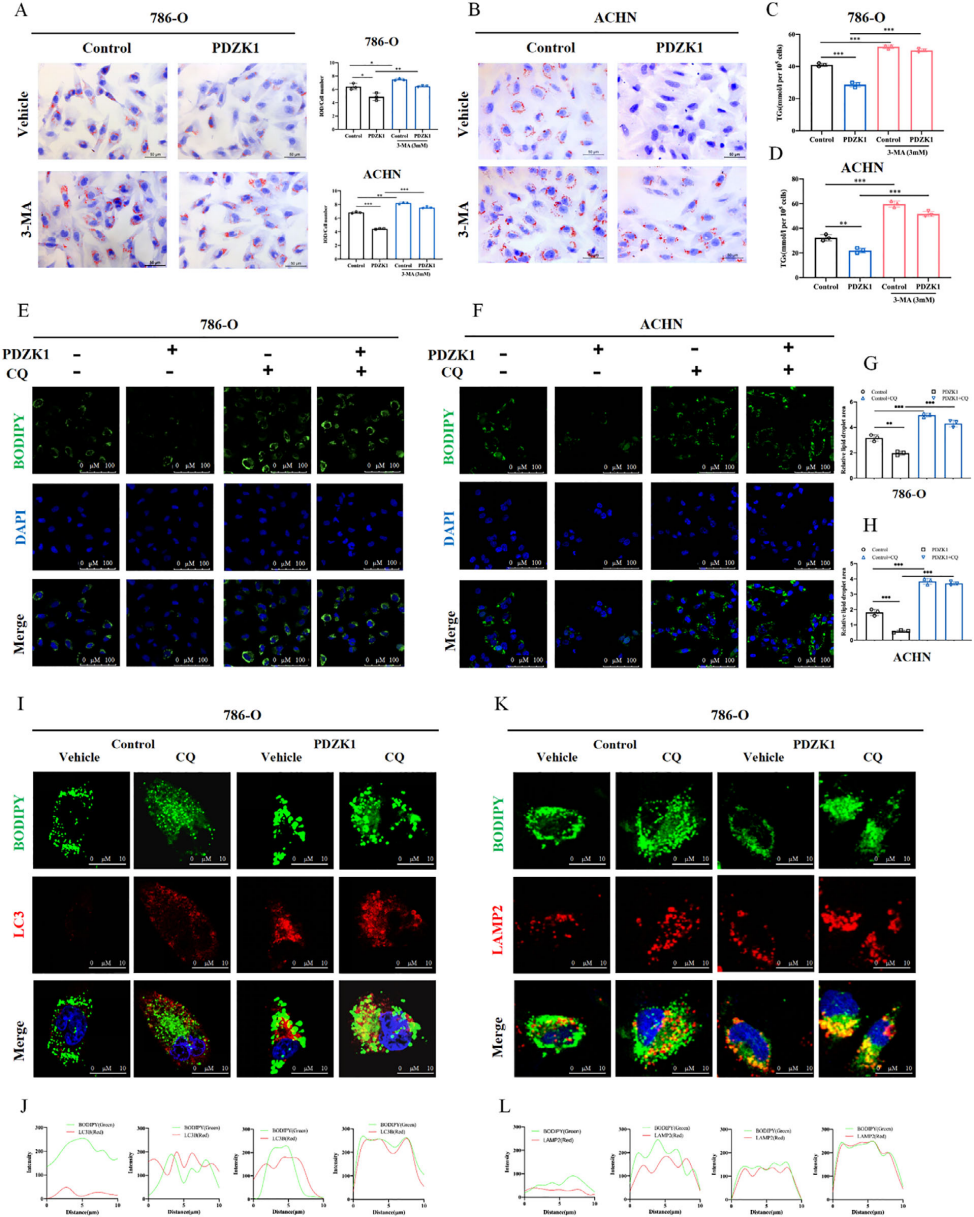
PDZK1 promotes autophagy‐dependent lipid droplet degradation. (A,B) Oil Red O staining of control and PDZK1 overexpressing (A) 786‐O and (B) ACHN cells treated with 3‐MA (2 mm, 24 h). Lipid droplets were quantified using ImageJ. Scale bar, 50 µm. Data are presented as mean ±SD (3 replicates). ^*^ *p* < 0.05, ^**^ *p *< 0.01, ^***^ *p* < 0.001. (C,D) Triglyceride (TG) levels measured in 786‐O cells with PDZK1 knockdown (C) or overexpression (D) after 24 h treatment with rapamycin or 3‐MA. Data are presented as mean ±SD (3 replicates). (E,F) BODIPY staining of lipid droplets in (E) 786‐O and (F) ACHN cells transfected with vector or PDZK1 overexpression, with or without chloroquine (CQ) treatment. Lipid droplets (green) were quantified using ImageJ. Scale bar, 100 µm. (G,H) Quantification of lipid droplet area per cell using ImageJ. Data are presented as mean ±SD. from three technical replicates. (I–L) Immunofluorescence staining of 786‐O cells treated with or without CQ for 24 h. Co‐staining was conducted for BODIPY (green) and LC3 (G, red) or LAMP2 (I, red). Colocalization statistics are shown in panels (H,J). Scale bar, 10 µm. In all statistical plots, data are shown as mean ± SD, one‐way ANOVA (Figure 3A,C,D,G,H) was used to determine statistical significance (^**^ *p* < 0.01, ^***^ *p *< 0.001).

To determine whether PDZK1 promotes lipophagy and thereby accelerates lipid droplet (LD) turnover in ccRCC cells, we overexpressed PDZK1 in 786‐O cells and treated the cells with 3‐methyladenine (3‐MA), which blocks autophagosome formation. Oil Red O staining showed that 3‐MA significantly reversed LD degradation induced by PDZK1 overexpression (Figure 3A,B). Consistently, triglyceride assays further confirmed that PDZK1 promotes LD degradation is dependent on a functional autophagy pathway (Figure 3C,D).

Lipophagy involves the sequestration of LDs by autophagosomes, followed by their degradation within lysosomes. To test whether lysosomal activity is required for PDZK1‐mediated LD degradation, we treated both 786‐O and ACHN cells overexpressing PDZK1 with chloroquine (CQ), a lysosomal inhibitor. CQ treatment prevented LD degradation in these cells (Figure 3E–H), indicating that lysosomal activity is essential for PDZK1‐mediated LD clearance.

We validated these findings using confocal microscopy to visualize the co‐localization of autophagosomes and LDs. Our findings revealed that the co‐localization between autophagosomes (labeled with LC3) and LDs (labeled with BODIPY) was enhanced in PDZK1‐overexpressing cells, indicating that PDZK1 facilitates the encapsulation of LDs by autophagosomes (Figure 3I,J; Figure S2D). Furthermore, enhanced co‐localization between LDs (labeled with BODIPY) and lysosomes (labeled with LAMP2) was also observed in these cells, suggesting that PDZK1 promotes LD degradation through the lysosomal pathway (Figure 3K,L). Together, these results demonstrate that PDZK1 facilitates autophagosome‐lysosome fusion, thereby promoting LD degradation via lipophagy.

### 2.4 PDZK1 Promotes Lipophagy by Upregulation of ULK1 Expression

**FIGURE 4:**
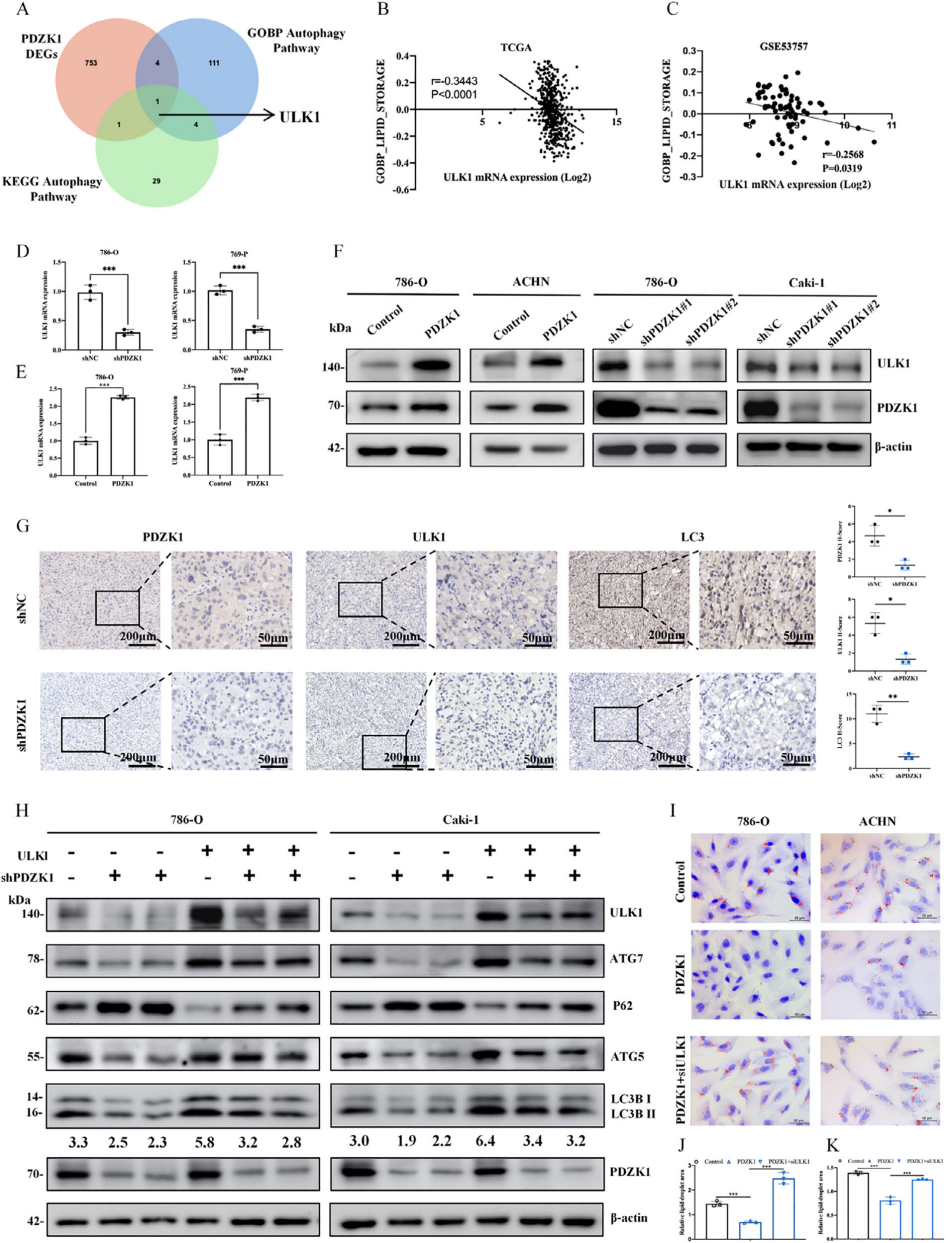
PDZK1 activates lipophagy by upregulating ULK1 expression. (A) The overlapped genes between differentially expressed genes and autophagy pathways. Differentially expressed genes are grouped by PDZK1 expression in TCGA database. The autophagy pathways are download from GO and KEGG database. (B, C) Negative correlation between the GSVA score of the lipid storage signature and ULK1 mRNA expression in the (B) TCGA and (C) GSE53757 datasets. (D,E) qPCR analysis of ULK1 mRNA expression. PDZK1 knockdown reduced ULK1 mRNA expression in 786‐O and 769‐P cells (D), whereas PDZK1 overexpression elevated ULK1 mRNA expression in 786‐O and 769‐P cells (E). Data represented mean ±SD from three independent replicates. (F) Western blot analysis showing that PDZK1 knockdown reduced ULK1 protein level in 786‐O and Caki‐1 cells, while PDZK1 overexpression elevated ULK1 protein level in 786‐O and ACHN cells. (G) Immunohistochemistry (IHC) staining of PDZK1, ULK1, and LC3 protein levels in control and PDZK1 knockdown orthotopic kidney tumors. (H) Western blot analysis demonstrating that ULK1 overexpression rescued PDZK1 knockdown‐induced inhibition of autophagy activation. (I–K) Oil Red O staining showing that ULK1 knockdown reversed the PDZK1 overexpression‐induced reduction in lipid storage (I,J). Scale bar, 50 µm. Lipid droplet quantification was performed using ImageJ (K). Data represent mean ±SD from three technical replicates. In all statistical plots, data are shown as mean ± SD, one‐way ANOVA (Figure 4J,K) and Two‐tailed unpaired Student's t test (Figure 4D,E,G) were used to determine statistical significance (^*^ *p* < 0.05, ^**^ *p *< 0.01, ^***^ *p *< 0.001).

To explore how PDZK1 promotes lipophagy in ccRCC cells, we integrated DEGs stratified by PDZK1 expression levels from the TCGA database with autophagy‐related gene sets from GO and KEGG databases. This analysis identified ULK1 as the unique overlapping gene (Figure 4A). Correlation studies showed a negative relationship between ULK1 expression and GSVA scores for LD accumulation in both TCGA and the independent GSE53757 cohort (Figure 4B,C). These results suggested that PDZK1 might regulate lipophagy through ULK1.

Functional analyses demonstrated that PDZK1 knockdown significantly suppressed both mRNA and protein levels of ULK1 in ccRCC cells. Conversely, PDZK1 overexpression increased ULK1 expression (Figure 4D–F). Consistent with these in vitro observations, in vivo studies using an orthotopic kidney tumor model revealed that PDZK1‐knockdown tumors exhibited reduced ULK1 expression and impaired autophagy activity relative to controls (Figure 4G). These results collectively indicate that PDZK1 upregulates ULK1 expression.

To clarify the functional role of the PDZK1‐ULK1 axis, we performed rescue experiments. Silencing ULK1 in PDZK1‐overexpressing cells abolished autophagy activation (Figure S3A); while ULK1 overexpression restored autophagy in PDZK1‐knockdown cells (Figure 4H). Oil Red O staining showed that PDZK1 overexpression enhanced LD degradation, an effect that was reversed by ULK1 knockdown (Figure 4I–K). Conversely, ULK1 overexpression reduced LD accumulation in PDZK1‐knockdown cells (Figure S3B–D).

In summary, these results demonstrate that the PDZK1‐ULK1 axis is essential for activating lipophagy and regulating LD dynamics in ccRCC cells.

### 2.5 LEF1 Mediates PDZK1‐Dependent Transcriptional Regulation of ULK1 in ccRCC

**FIGURE 5:**
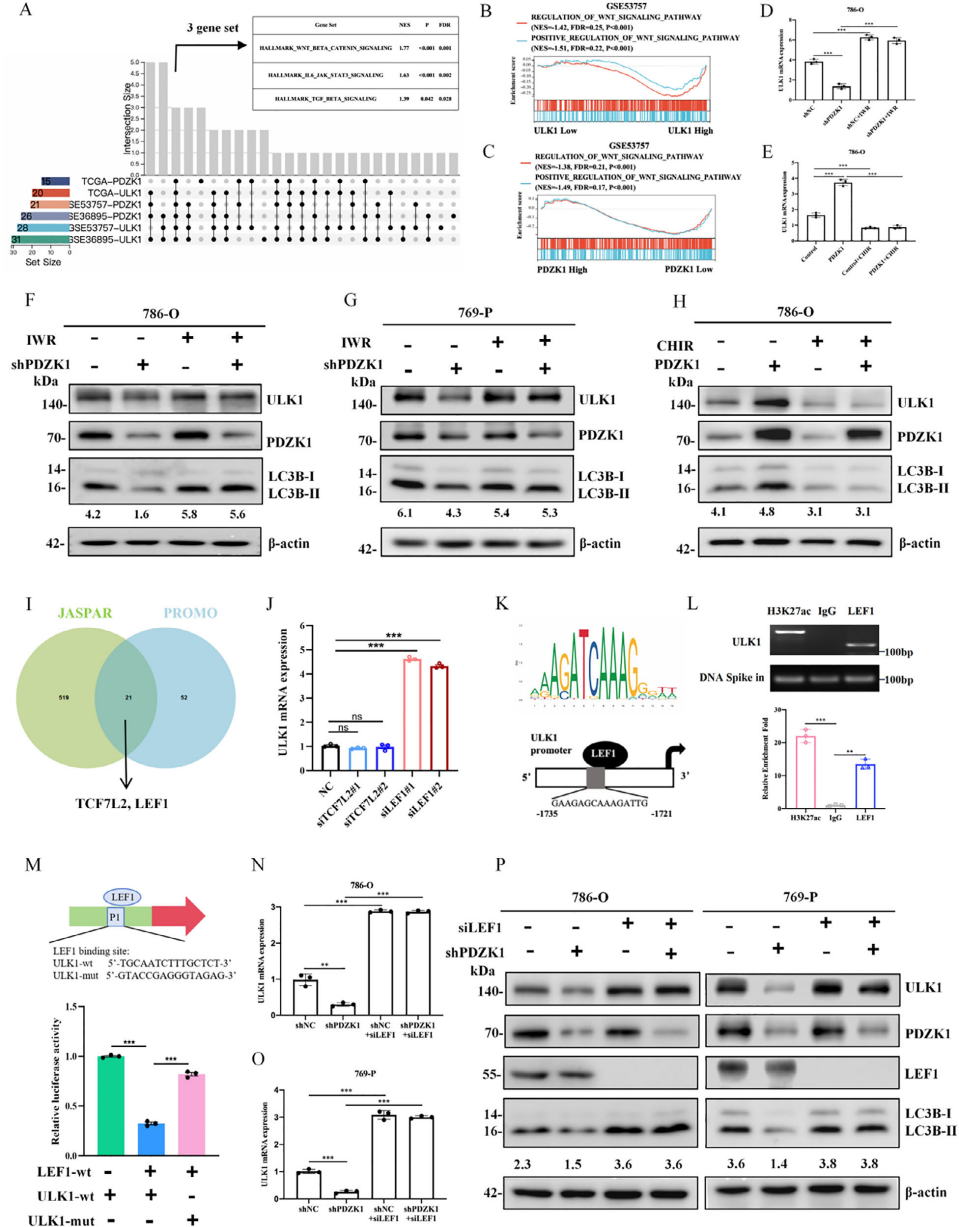
LEF1 mediates the transcription regulation of ULK1 in PDZK1‐dependent manner. (A) Identification of overlapping pathways by GSEA based on PDZK1 and ULK1 expression in the TCGA, GSE53757, and GSE36895 datasets. (B,C) GSEA enrichment plots showing significant association of the Wnt/β‐catenin pathway with (B) low ULK1 expression or (C) low PDZK1 expression in ccRCC specimens from the GSE53757 datasets. (D) Inhibition of Wnt/β‐catenin pathway elevated ULK1 mRNA expression, reversing the suppression caused by PDZK1 knockdown in 786‐O cells. (E) Activation of the Wnt/β‐catenin pathway decreased ULK1 mRNA expression, reversing the elevation caused by PDZK1 overexpressing in 786‐O cells. (F,G) Inhibition of Wnt/β‐catenin pathway activation restored ULK1 protein levels suppressed by PDZK1 knockdown in 786‐O and 769‐P cells. (H) Activation of the Wnt/β‐catenin pathway reduced ULK1 protein levels elevated by PDZK1 overexpressing in 786‐O cells. (I) Overlapping transcription factors predicted to bind the ULK1 promoter region, identified using the PROMO and JASPAR databases. (J) qPCR analysis of ULK1 mRNA expression in 786‐O cells transfected with siRNA targeting LEF1 or TCF7L2. (K) JASPAR analysis results for LEF1 binding sites located within the promoter of ULK1 gene. (L) CUT & Tag‐qPCR evaluation in 786‐O cells revealed elevated degrees of LEF1 adhesion compared to negative control. (M) Confirmation of the binding between LEF1 and the ULK1 promoter using a dual‐luciferase reporter assay. (N,O) Knockdown of LEF1 elevated ULK1 mRNA levels in (N) 786‐O and (O) 769‐P cells, both with and without PDZK1 knockdown, as determined by qPCR analysis. (P) Knockdown of LEF1 also elevated ULK1 protein expression in 786‐O and 769‐P cells, both with and without PDZK1 knockdown, as shown by Western blot analysis. In all statistical plots, data are shown as mean ± SD (3 replicates), one‐way ANOVA (Figure 5D,E,J,L–O) was used to determine statistical significance (ns = not significant, ^*^ *p* < 0.05, ^**^ *p* < 0.01, ^***^ *p* < 0.001).

To elucidate the mechanisms underlying PDZK1‐dependent regulation of ULK1 in ccRCC cells, we performed GSEA analysis across multiple cohorts, including TCGA, GSE36895, and GSE53757. Patients were grouped based on PDZK1 or ULK1 mRNA expression levels. UpSetR analysis identified the Wnt/β‐catenin signaling pathway as the most significantly overlapping pathway across these groups (Figure 5A). Notably, this pathway was enriched in both low‐PDZK1 and low‐ULK1 expression groups (Figure 5B,C), suggesting that Wnt/β‐catenin signaling modulates ULK1 expression through PDZK1.

To test this hypothesis, we treated cells with Wnt/β‐catenin signaling modulators, including IWR‐1‐endo (an inhibitor) and CHIR‐98014 (an activator). IWR‐1‐endo treatment reversed the reduction of ULK1 mRNA expression caused by PDZK1 knockdown (Figure 5D), while CHIR‐98014 attenuated ULK1 upregulation in PDZK1‐overexpressing cells (Figure 5E). Western blotting confirmed these findings at the protein level. Autophagy flux assays further demonstrated corresponding alterations in LC3B‐II/LC3B‐I ratio, supporting the functional association between Wnt/β‐catenin signaling and ULK1‐dependent autophagy (Figure 5F–H; Figure S4A).

To identify potential transcriptional effector regulating ULK1 expression through Wnt/β‐catenin pathway, we performed bioinformatics analysis using the PROMO and JASPAR databases (Table S2). This analysis identified 21 candidate transcription factors with predicted binding sites in the ULK1 promoter region (Figure 5I). FIMO analysis revealed that LEF1 ranked nineth with a significant motif match score and p‐value, while TCF7L2 ranked 19th with lower binding prediction scores (Table S3). Functional validation demonstrated that LEF1 knockdown increased ULK1 mRNA expression, while TCF7L2 knockdown had no effect (Figure 5J), confirming LEF1 as the functional transcriptional regulator of ULK1 in ccRCC. Given the established role of LEF1 in β‐catenin‐dependent transcriptional regulation [18], we further investigated whether LEF1 directly regulates ULK1 transcription.

ChIP‐seq data analysis identified two binding peaks for LEF1: one approximately 1.7 kb upstream and another 50 bp downstream of the ULK1 transcription start site (Figure S4B). CUT&Tag‐qPCR assays confirmed LEF1 binding to these promoter regions in 786‐O cells (Figure S4C). Motif analysis using JASPAR revealed a consensus sequence (GAAGAGCAAAGATTG) within peak 1 (Figure 5K), with approximately 2‐fold enrichment in LEF1‐bound chromatin (Figure 5L). These results establish LEF1 as a direct transcriptional repressor of ULK1 in ccRCC. To further validate this mechanism, dual‐luciferase reporter assays were performed using wild‐type and P1 site‐mutant constructs based on the LEF1 binding site identified by CUT&Tag‐qPCR. Co‐transfection of LEF1 with the wild‐type reporter significantly reduced luciferase activity, whereas it failed to affect the P1‐mutant reporter (Figure 5M), confirming that LEF1 directly represses ULK1 transcription by binding to the P1 site within the ULK1 promoter.

Functional validation of the PDZK1/LEF1/ULK1 axis showed that LEF1 knockdown rescued ULK1 expression and autophagy flux in PDZK1‐depleted cells (Figure 5N–P). LD accumulation assays revealed that PDZK1 knockdown increased LD accumulation, which was reversed by LEF1 silencing (Figure S4D). Notably, dual knockdown of LEF1 and ULK1 significantly enhanced LD accumulation compared to LEF1 knockdown alone (Figure S4E). These findings demonstrate the critical role of LEF1 in mediating PDZK1‐dependent regulation of ULK1 and its impact on lipophagy.

### 2.6 PDZK1 Suppresses LEF1 Nuclear Translocation Through Direct Interaction

**FIGURE 6:**
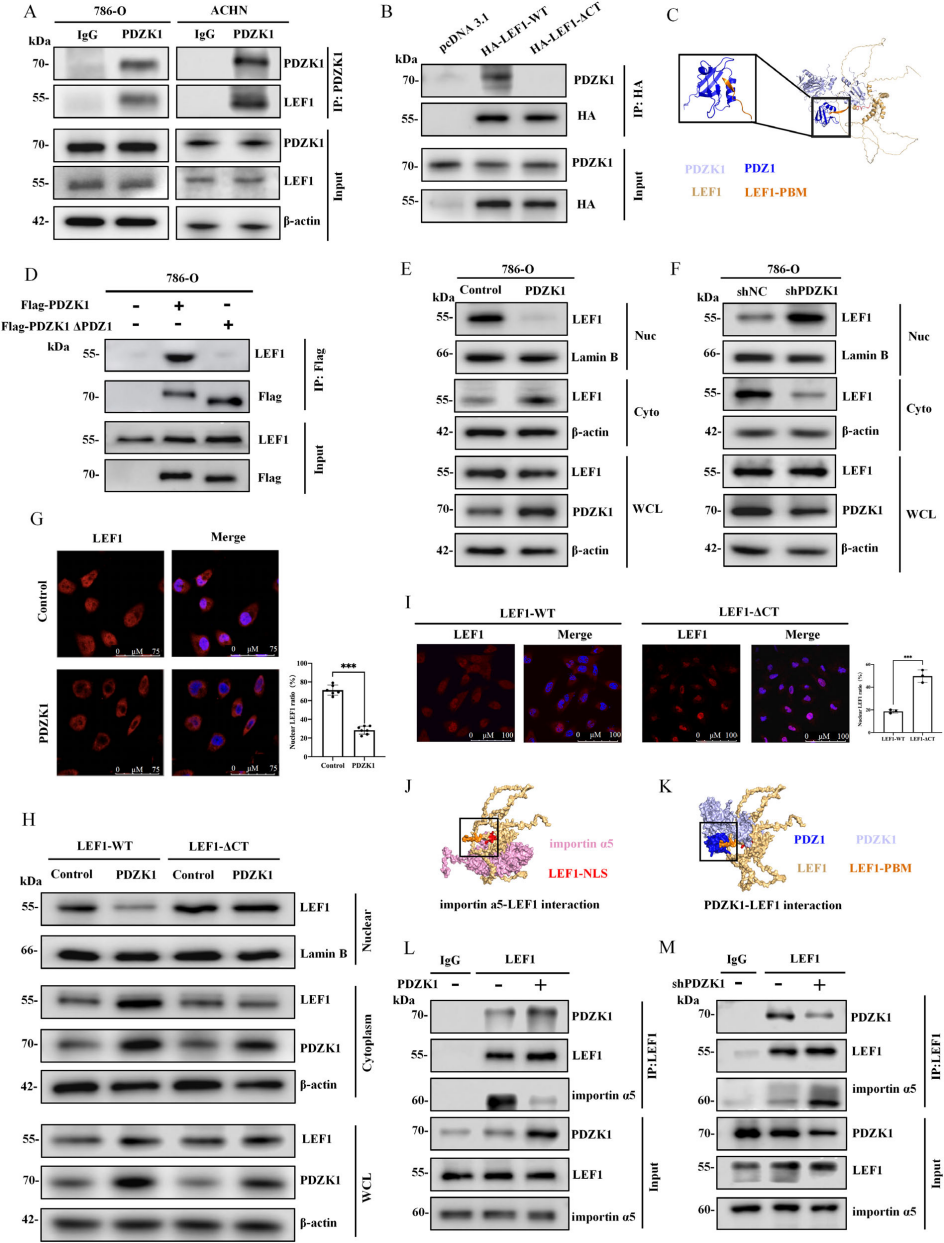
PDZK1 inhibits LEF1 nuclear translocation by binding to LEF1. (A) Endogenous interaction between PDZK1 and LEF1 in 786‐O and ACHN cell lines was demonstrated by Co‐IP. (B) Co‐IP assay confirmed that PDZK1 binds to LEF1‐WT but not LEF1‐ΔCT in transfected 786‐O cells. (C) Molecular docking model illustrated the binding interface between PDZK1 and LEF1. (D) Co‐IP assay confirmed that LEF1 binds to PDZK1 but not PDZK1‐ΔPDZ1 in transfected 786‐O cells. (E) Western blot analysis of nuclear and cytosolic fractions from control and PDZK1 overexpressing 786‐O cells. (F) Western blot analysis of nuclear and cytosolic fractions from control and PDZK1 knockdown 786‐O cells. (G) Immunofluorescence assay visualizing the subcellular localization of LEF1 in control and PDZK1 overexpressing 786‐O cells. (H) Subcellular fractionation analysis of LEF1 expression in control and PDZK1 overexpressing cells transfected with HA‐LEF1‐WT or HA‐LEF1‐ΔCT. (I) Immunofluorescence assay showing the subcellular localization of LEF1 in 786‐O cells co‐transfected with PDZK1 and HA‐LEF1‐WT or HA‐LEF1‐ΔCT. (J) The prediction of the interaction between importin α5 and LEF1 was performed using H‐DOCK and visualized on the surface using PyMOL. (K) Prediction of the interaction between PDZK1 and LEF1 performed using H‐DOCK with PyMOL visualization. (L,M) Co‐IP assay revealing the association of importin α5 with LEF1 in control and PDZK1 overexpressing 786‐O cells (L) or control and PDZK1 knockdown 786‐O cells (M).

To investigate the role of PDZK1 in regulating LEF1‐mediated ULK1 expression, we explored the potential interaction between PDZK1 and LEF1. PDZK1, a scaffold protein containing PDZ domains, modulates cellular signaling by interacting with target proteins. Notably, the C‐terminus region of LEF1 contains a conserved type II PDZ‐binding motif (AAYI), suggesting a possible interaction with PDZK1. Co‐immunoprecipitation (Co‐IP) assays confirmed that LEF1 directly binds to PDZK1 (Figure 6A; Figure S5A), with the C‐terminal region of LEF1 being essential for this interaction (Figure 6B; Figure S5B). Protein structure modeling and docking studies provided structural insights into the PDZK1‐LEF1 complex (Figure 6C). Further validation using a PDZ1‐deleted PDZK1 mutant (PDZK1‐ΔPDZ1) demonstrated that the PDZ1 domain of PDZK1 specifically interacts with the LEF1 C‐terminus, thereby validating the accuracy of our molecular docking predictions (Figure 6D).

LEF1, a transcription factor of the TCF/LEF family, translocates to the nucleus by importin α5 via its nuclear localization sequence (NLS) [19]. Overexpression of PDZK1 reduced nuclear LEF1 levels (Figure 6E; Figure S5C), while PDZK1 knockdown increased nuclear LEF1 accumulation (Figure 6F; Figure S5D). Importantly, total LEF1 protein levels remained unchanged, suggesting that PDZK1 influences the subcellular distribution of LEF1 rather than its overall expression. Immunofluorescence assays confirmed that PDZK1 sequestered LEF1 in the cytoplasm (Figure 6G). Consistent with these findings, immunohistochemical analysis of ccRCC tissue microarrays revealed an inverse correlation between PDZK1 expression and nuclear LEF1 localization (Figure S5E).

To determine whether the PDZK1‐LEF1 interaction blocks nuclear translocation, we overexpressed wild‐type LEF1 or a C‐terminal mutant lacking the PDZ‐binding motif in 786‐O cells. Wild‐type LEF1 bound to PDZK1, suppressing its nuclear import. In contrast, the C‐terminal mutant LEF1 failed to interact with PDZK1 and exhibited unregulated nuclear accumulation (Figure 6H). Immunofluorescence analysis further validated this finding (Figure 6I). Functionally, the cytoplasmic retention of LEF1 mediated by PDZK1 led to diminished ULK1 transcription and impaired autophagy activity (Figure S5F). To assess the functional relevance of the PDZK1‐LEF1 interaction in vivo, we employed xenograft models and observed that PDZK1 overexpression significantly suppressed tumor growth. Importantly, this tumor‐suppressive effect was abrogated upon co‐expression of LEF1‐ΔCT, confirming that the tumor‐suppressive function of PDZK1 is mediated through its directly interacting with LEF1 (Figure S5G–I).

Mechanistically, protein‐protein docking simulations suggested that PDZK1 binding sterically obstructs the interaction between LEF1 and importin α5 (Figure 6J,K). Co‐IP assays confirmed this mechanism, showing that PDZK1 knockdown promotes LEF1‐importin α5 complex formation, whereas PDZK1 overexpression disrupts this interaction (Figure 6L,M).

Collectively, these results demonstrate that PDZK1 acts as a cytoplasmic anchor for LEF1, preventing its nuclear translocation and inhibiting LEF1‐dependent ULK1 transcription.

### 2.7 The PDZK1‐ULK1 Axis Suppresses ccRCC Progression via Lipophagy Activation

**FIGURE 7:**
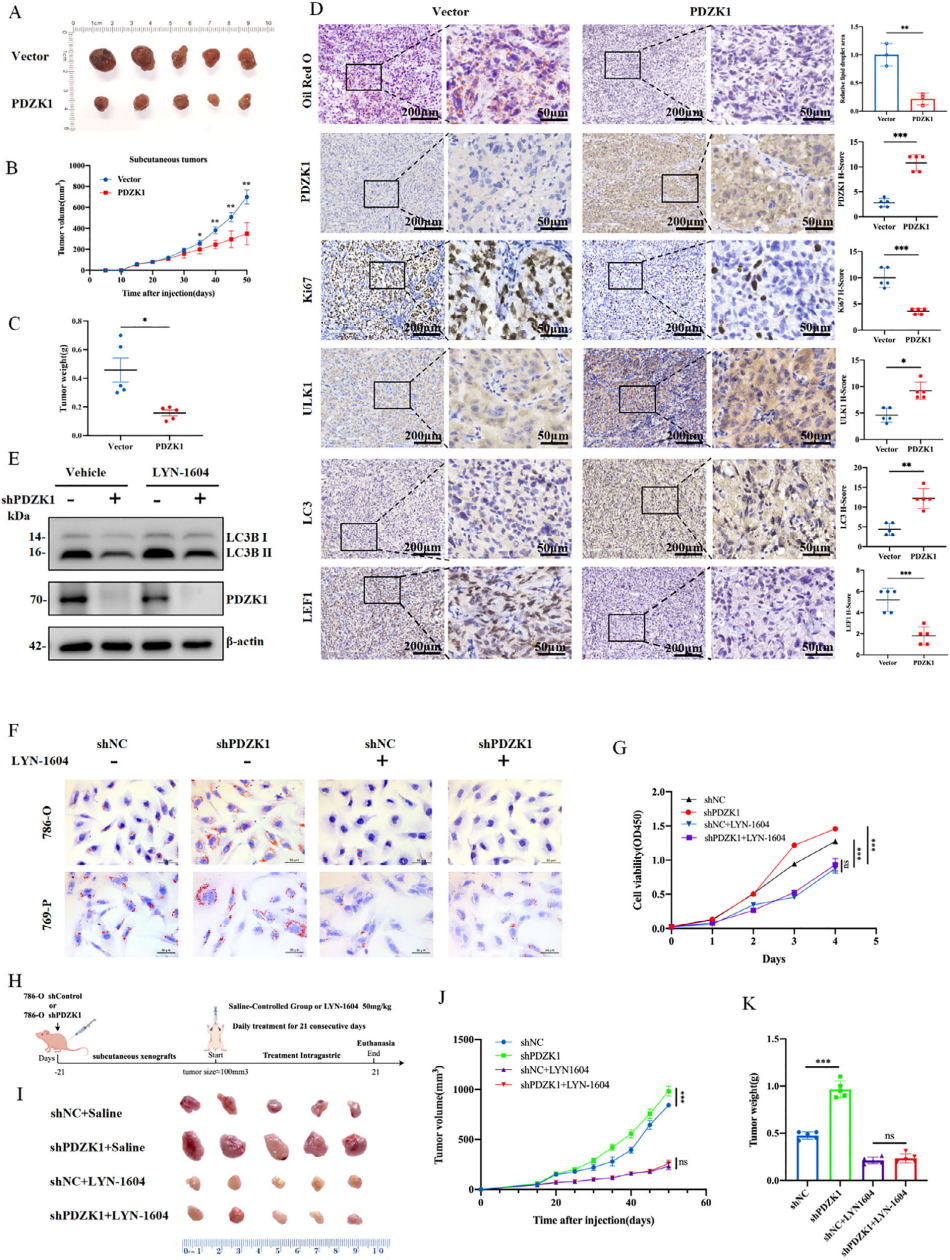
The PDZK1‐ULK1 axis inhibits tumor progression by activating lipophagy. (A–C) Xenograft assay of control or PDZK1‐overexpressing 786‐O cells in nude mice. Tumor volume was monitored regularly (B), and tumors were weighed after sacrifice (C). Data are presented as mean ± SD (5 replicates). (D) Oil Red O staining and IHC staining for PDZK1, LC3, Ki67, LEF1, and ULK1 in xenografted tumors. Representative images from five independent experiments are shown. For visualization, brightness and contrast were uniformly adjusted across all groups. All quantitative analyses were performed on the original, unprocessed images. Magnified views of dashed areas are shown in the right panels. Scale bars, 100 µm. H‐score was calculated and presented as mean ± SD (5 replicates). (E) Western blot analysis of autophagy activity in control and PDZK1 knockdown 786‐O cells treated with or without 4 µm LYN‐1604 for 24 h. (F) Oil Red O staining of the control or PDZK1 knockdown 786‐O cells with LYN‐1604 treatment. Scale bar, 50 µm. (G) CCK‐8 assay of control and PDZK1 knockdown 786‐O cells treated with LYN‐1604, assessing cell viability. Data were expressed as mean ± SD (3 replicates). (H) Schematic of animal experiment design. (I) Representative images of subcutaneous tumors from shControl + Saline, shPDZK1 + Saline, shControl + LYN‐1604, and shPDZK1 + LYN‐1604 groups in nude mice. (J,K) Xenograft assay of control or PDZK1‐knockdown 786‐O cells treated with LYN‐1604 or saline in nude mice. The tumor size was monitored every 4 days, with the last measurement performed on day 50 (J). Tumors were harvested and weighed after mice were euthanized (K). Data are presented as mean ± SD (5 replicates). In all statistical plots, data are shown as mean ± SD, one‐way ANOVA (Figure 7G,J,K) and Two‐tailed unpaired Student's t test (Figure 7B–D) were used to determine statistical significance (*ns* = not significant, ^*^ *p* < 0.05, ^**^ *p *< 0.01, ^***^ *p *< 0.001).

Abnormal LD accumulation is a key driver of tumor growth in ccRCC [20]. To elucidate the tumor‐suppressive role of PDZK1 and its potential as a therapeutic target, we established subcutaneous tumor models. Our results showed that PDZK1 overexpression significantly reduced tumor volume and weight (Figure 7A–C). Immunohistochemistry (IHC) analysis showed increased LC3 and ULK1 expression levels, along with reduced nuclear LEF1 expression and LD accumulation in PDZK1‐overexpressing tumors (Figure 7D). Together, these results indicate that PDZK1‐mediated lipophagy plays a critical role in suppressing ccRCC progression in vivo.

To investigate the functional contribution of ULK1, we treated ccRCC cells with LYN‐1604, a selective ULK1 agonist [21]. In PDZK1‐knockdown cells, LYN‐1604 treatment restored autophagy flux, as indicated by an increased LC3B‐II/LC3B‐I ratio (Figure 7E). Moreover, LYN‐1604 treatment markedly reduced LD accumulation (Figure 7F; Figure S6A) and significantly inhibited cell viability in both control and PDZK1‐knockdown cells (Figure 7G). These findings suggest ULK1 as a critical effector of PDZK1's tumor‐suppressive function.

To validate the PDZK1‐ULK1 axis in vivo, we administered LYN‐1604 to subcutaneous tumor models. Tumors from both control and PDZK1‐knockdown groups exhibited similar reductions in volume and weight following LYN‐1604 treatment (Figure 7H–K). Oil Red O staining confirmed enhanced LD degradation (Figure S6B), and IHC analysis demonstrated the upregulation of ULK1 expression (Figure S6C). Collectively, these findings reveal a novel therapeutic mechanism that PDZK1 promotes ULK1‐dependent lipophagy to degrade oncogenic LDs, thereby suppressing ccRCC progression.

### 2.8 ULK1 Agonist Sensitizes PDZK1 Deficiency‐Induced High LD Accumulation Tumors to Sunitinib

**FIGURE 8:**
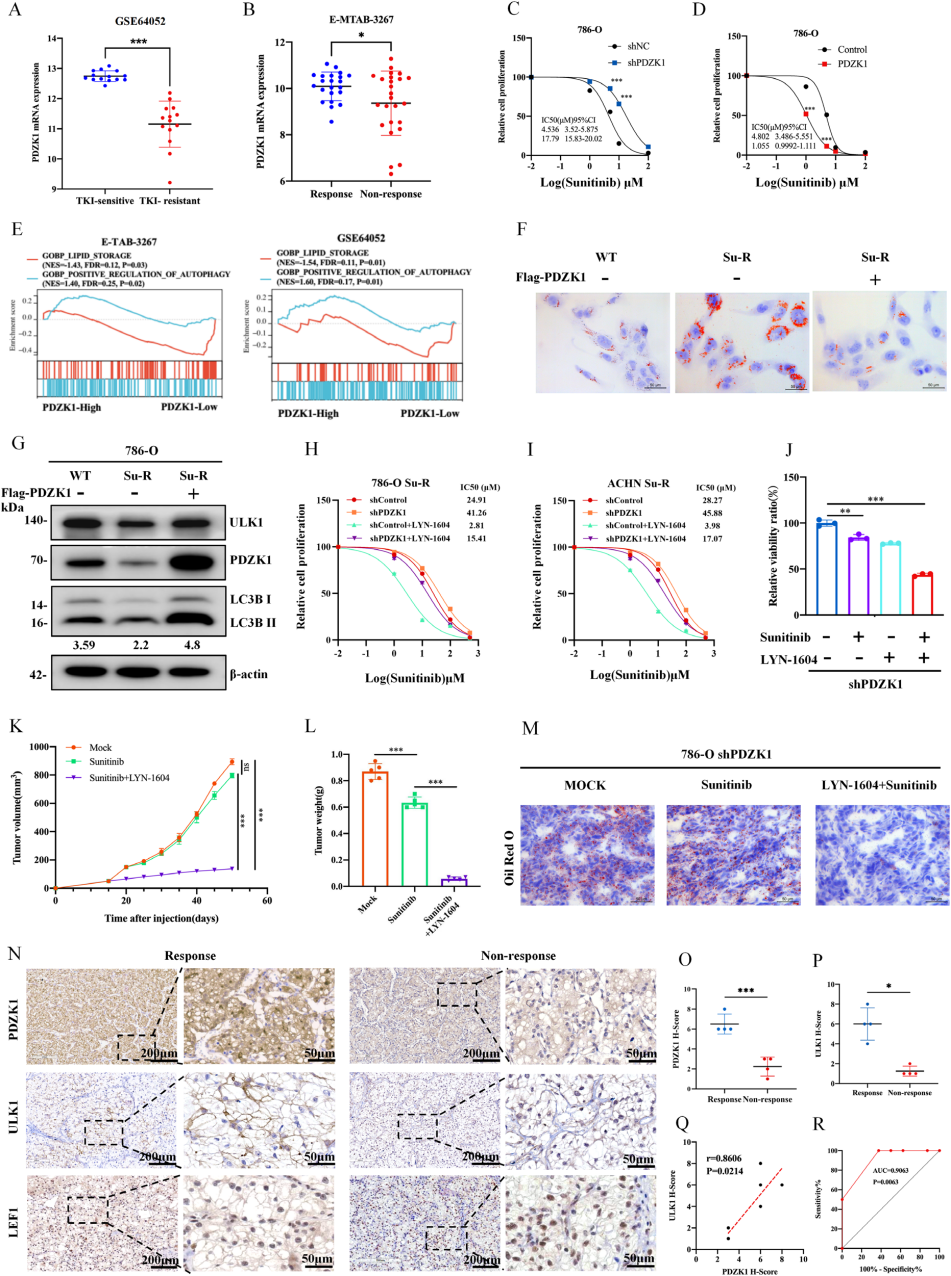
ULK1 agonist sensitizes PDZK1 deficiency‐induced high LDs accumulation tumors to sunitinib. (A) PDZK1 mRNA expression was elevated in patients with good response to sunitinib than in poor response patients from the GSE64052 dataset. Data were presented as mean ± SD. ^***^ *p* < 0.001. (B) PDZK1 mRNA expression was downregulated in sunitinib‐resistant ccRCC xenografts from the E‐MTAB‐3267 dataset. Data were presented as mean ± SD. ^*^ *p* < 0.05. (C) PDZK1 knockdown increased sunitinib IC50 in 786‐O cells. Dose‐response survival curves were generated after 48 h of sunitinib treatment. Data are presented as mean ± SD, 3 replicates. (D) PDZK1 overexpression reduced sunitinib IC50 in 786‐O cells. Dose‐response survival curves were generated after 48 h of sunitinib treatment. Data are presented as mean ± SD, 3 replicates. (E) GSEA enrichment analysis of sunitinib‐resistant ccRCC specimens in the E‐MTAB‐3267 and GSE64052 datasets showed enrichment of autophagy pathways in those with high PDZK1 expression and lipid droplet pathways in specimens with low PDZK1 expression. (F) Oil red O staining in sunitinib‐sensitive or sunitinib‐resistant 786‐O cells, with resistant cells were transfected with Flag‐PDZK1. (G) Western blot analysis of ULK1, PDZK1, and LC3B in sunitinib‐sensitive or sunitinib‐resistant 786‐O cells, with resistant cells were transfected with Flag‐PDZK1. (H,I) PDZK1‐ULK1 axis increased sunitinib sensitivity in sunitinib‐resistant RCC cells. (H) 786‐O Su‐R or (I) ACHN Su‐R cells were transfected with shControl or shPDZK1 and treated with or without the ULK1 agonist LYN‐1604. Dose‐response curves demonstrate relative cell proliferation following sunitinib treatment. IC50 values (µM) are indicated in the legend. (J) CCK8 assay of PDZK1 knockdown 786‐O cells treated with vehicle, 2 µm suntininib, 8 µm LYN‐1604, or 2 µm suntininib + 8 µm LYN‐1604. Data were expressed as mean ± SD, 3 replicates. (K,L) Tumor volume was monitored every 5 days, with the last measurement performed on day 50 (K). Tumors were harvested and weighed after mice were euthanized (L). Data were presented as the mean ± SD, 5 replicates. (M) Photomicrographs of Oil red O staining of tumor xenografts from shPDZK1 + vehicle, shPDZK1 + LYN‐1604, or shPDZK1 + LYN‐1604+ sunitinib groups. (N) IHC analysis of PDZK1, ULK1, and LEF1 expression in specimens from ccRCC patients with response(R) or non‐response (NR) to sunitinib. scale bars, 200 µm. Right panels are magnification of the dashed areas on the left. scale bars, 100 µm. (O,P) Dot plot showing the quantification of PDZK1(N) or ULK1(O) H‐score in ccRCC specimens. Data were presented as the mean ± SD, 5 replicates. (Q) Correlation analysis of PDZK1 H‐score and ULK1 H‐score in ccRCC specimens. (R) ROC curve of ULK1 H‐score in ccRCC specimens, supporting its potential as a predictive marker for TKI resistance. In all statistical plots, data are shown as mean ± SD, one‐way ANOVA (Figure 8H,K,L) and Two‐tailed unpaired Student's t test (Figure 8A–D,O,P) were used to determine statistical significance (ns = not significant, ^*^ *p* < 0.05, ^**^ *p* < 0.01, ^***^ *p* < 0.001).

LD accumulation is a well‐established driver of sunitinib resistance in ccRCC [22]. To determine whether PDZK1 enhances sunitinib sensitivity by inhibiting LD accumulation, we analyzed the correlation between PDZK1 expression and clinical response to sunitinib. In ccRCC patients, low PDZK1 expression was linked to a poor response to sunitinib (Figure 8A,B). In vitro, PDZK1 knockdown significantly increased the IC50 of sunitinib, indicating reduced sensitivity, while overexpression of PDZK1 restored sunitinib sensitivity by lowering the IC50 (Figure 8C,D). GSEA further demonstrated that high PDZK1 expression correlated with autophagy activation and reduced LD storage in sunitinib‐resistant patients (Figure 8E).

We next established a sunitinib‐resistant ccRCC cell line (Figure S7A), SU‐R‐786O, which exhibited increased LD accumulation, decreased PDZK1 and ULK1 expression, and impaired autophagy flux (Figure 8F,G). Overexpressing PDZK1 in SU‐R‐786O cells significantly reduced LD accumulation and restored both ULK1 expression and autophagic flux (Figure 8F,G).

Given that PDZK1 knockdown inhibits lipophagy and promotes LD accumulation, we hypothesized that activating lipophagy could overcome sunitinib resistance. To activate lipophagy, we treated cells with the ULK1 agonist LYN‐1604, which re‐sensitized both PDZK1‐knockdown and sunitinib‐resistant 786‐O cells to sunitinib (Figure S7B,C). To confirm the role of the PDZK1‐ULK1 axis, we tested whether ULK1 activation could rescue sunitinib resistance in PDZK1‐knockdown cells. Treatment with a ULK1 agonist restored sunitinib sensitivity in both 786‐O and ACHN sunitinib‐resistant cells with PDZK1 knockdown, demonstrating that ULK1 functions downstream of PDZK1 in mediating sunitinib sensitivity (Figure 8H,I). Notably, monotherapies with either sunitinib or LYN‐1604 had limited inhibitory effects, their combination synergistically inhibited cell viability (Figure 8J). These findings indicate that LYN‐1604 enhances sunitinib sensitivity by promoting lipophagy.

We further evaluated the efficacy of this combination in vivo (Figure S7D). In a PDZK1‐deficient ccRCC model, tumors treated with sunitinib alone showed no significant reduction in volume or weight compared to controls. However, combined treatment with LYN‐1604 and sunitinib significantly suppressed tumor growth (Figure 8K,L). This combination reduced LD accumulation (Figure 8M), increased ULK1 and LC3 expression levels, and decreased Ki67‐positive cells, indicating enhanced lipophagy activation (Figure S7E).

To explore the clinical relevance of PDZK1 and ULK1 expression in sunitinib response, we examined their levels in a cohort of ccRCC patients. IHC staining showed higher PDZK1 and ULK1 expression, along with reduced nuclear LEF1, in sunitinib responders compared to non‐responders (Figure 8N–P). Pearson correlation analysis revealed a strong positive association between PDZK1 and ULK1 protein levels (Figure 8Q). Furthermore, ROC analysis of ULK1 expression indicated a high predictive value for sunitinib response, with an AUC of 0.9063 (Figure 8R).

Together, our findings demonstrate that PDZK1 deficiency inhibits ULK1‐mediated lipophagy, leading to sunitinib resistance in ccRCC. The combined treatment with the ULK1 agonist LYN‐1604 effectively overcomes sunitinib resistance, providing a novel therapeutic strategy to enhance sunitinib efficacy (Figure S7E).

## 3 Discussion

Metabolic reprogramming is a prominent feature of ccRCC, manifested by excessive LD accumulation. Intracellular LDs play a crucial role in ccRCC progression and sunitinib resistance by providing an energy source, supporting membrane biosynthesis, alleviating endoplasmic reticulum stress, and preventing lipotoxicity [6, 8, 9, 23]. However, the mechanism of regulating LD accumulation in ccRCC cells is still not fully understood.

In this study, we observed that reduced PDZK1 expression correlates with increased LD accumulation and poor prognosis in ccRCC, based on data from multiple independent cohorts. Integrated analysis of scRNA‐Seq and bulk RNA‐Seq data further revealed a positive correlation between PDZK1 expression and LD degradation specifically in epithelial (tumor) cell subpopulations from ccRCC patients (Figure 1A–D). These results were validated through functional experiments in ccRCC cells, as well as in subcutaneous tumors and orthotopic kidney tumor models (Figures 1E–M and 7A–D). To our knowledge, this is the first study to establish that PDZK1 promotes LD degradation, providing a new insight into lipid metabolism regulation in ccRCC.

Promoting LD degradation is a critical strategy for reducing lipid accumulation in ccRCC. Previous studies have reported that monoglyceride lipase mitigates LD accumulation by activating lipolysis [24]. Notably, our study reveals a previously unexplored autophagy‐dependent pathway for LD degradation. By stratifying ccRCC patients based on LD degradation scores, we found that patients with activated LD degradation exhibited significant enrichment in autophagy pathways, while no significant enrichment was observed in lipolysis pathways, highlighting the pivotal role of autophagy in LD degradation in ccRCC. Furthermore, patients with high PDZK1 expression were also enriched in autophagy pathways. These findings suggest that PDZK1 may promote LD degradation in ccRCC by activating autophagy (Figure 2A,B).

To explore this hypothesis, we employed Western blotting, mCherry‐GFP‐LC3 adenovirus, and transmission electron microscopy to confirm that PDZK1 activates autophagy in ccRCC cells (Figure 2). Our experiments revealed that PDZK1 expression promoted the co‐localization of autophagosomes with LDs, promoting their subsequent fusion with lysosomes, indicating that PDZK1 facilitates autophagic LD turnover (Figure 3). These findings reveal a novel autophagy‐dependent pathway for LD degradation in ccRCC cells and highlight the role of PDZK1‐mediated lipophagy in alleviating LD accumulation.

To elucidate the molecular mechanism by which PDZK1 regulates lipophagy, we identified ULK1 as the only autophagy‐related DEG associated with PDZK1. Notably, we observed that ULK1, essential for autophagosome formation and autophagy activation, was upregulated in responders compared to sunitinib‐resistant patients (Figure 8N–P), suggesting its potential as a predictive biomarker for sunitinib response (AUC = 0.9063, p = 0.0063).

We then explored the regulatory mechanism of ULK1 in ccRCC and found that PDZK1 upregulates ULK1 expression both in vitro and in vivo, establishing the PDZK1‐ULK1 axis as a novel driver of autophagy‐dependent LD degradation in ccRCC (Figure 4). Our investigation further found that the inhibition of Wnt/β‐catenin pathway mediates the upregulation of ULK1 by PDZK1 (Figure 5A–H). By integrating computational predictions from JASPAR and PROMO with experimental validation via CUT&Tag‐qPCR and dual‐luciferase reporter assays, we demonstrated that LEF1, a core transcription factor in the Wnt/β‐catenin pathway, directly binds to the ULK1 promoter and represses its transcription (Figure 5I–M). This study elucidated a novel mechanism of ULK1 transcriptional repression, highlighting the interplay between Wnt/β‐catenin pathway and autophagy regulation.

PDZ domain‐containing proteins can interact with transcription factors to regulate their stability, nuclear translocation, or transcription activity. It has been reported that PDLIM2 inhibits STAT3 nuclear translocation and transcriptional activity by directly binding [25]. Similarly, Erbin specifically interacts with Smad2/Smad3 to inhibit TGFβ signaling [26]. In our study, we identified a class II PDZ domain‐binding motif (A‐A‐Y‐I) in the C‐terminal region of LEF1, which interacts with PDZK1. Through Co‐IP assay and molecular docking, we confirmed that PDZK1 directly interacts with LEF1 (Figure 6A–D). This interaction inhibits LEF1's nuclear translocation and transcription activity, thereby suppressing ULK1 expression and autophagy activity (Figure 6E–I). Our study elucidates a novel molecular mechanism by which PDZK1, functioning as a scaffold protein, inhibits LEF1 nuclear translocation and transcription activity.

Sunitinib, a multi‐receptor tyrosine kinase inhibitor, is a first‐line treatment for renal cell carcinoma. However, 20%‐30% of renal cell carcinoma patients exhibit primary resistance to sunitinib, while the remaining patients develop resistance within 6–15 months of treatment [27, 28, 29]. Resistance mechanisms include the upregulation of alternative pro‐angiogenic pathways, activation of epithelial‐mesenchymal transition (EMT), and regulation by miRNAs [30]. Recently, excessive LD accumulation has been implicated in sunitinib resistance in ccRCC [31, 32, 33]. Therapies targeting lipid metabolism in ccRCC could improve patient prognosis and overcome sunitinib resistance. Although CK2 inhibitors have exhibited synergistic anti‐tumor effects with sunitinib through reducing fatty acid synthesis in ccRCC [34], the potential of targeting LD degradation remains underexplored. In this study, we demonstrate that LYN‐1604, a small‐molecule agonist of ULK1, promoted LD degradation by enhancing autophagy in ccRCC cells (Figure 7E–G). Notably, we treated subcutaneous mouse models with LYN‐1604, observing elevated autophagy‐related protein expression in both control and PDZK1 knockdown groups. Consistently, the tumor growth and LD accumulation were significantly suppressed after treatment (Figure 7H–K), confirming that the PDZK1‐ULK1 axis inhibits tumor progression via activating lipophagy, providing a new perspective for clinical treatment.

We observed reduced PDZK1 and ULK1 expression with increased LD accumulation in sunitinib‐resistant cells. Restoring PDZK1 expression reactivated ULK1 and autophagy, reducing LD accumulation and enhancing sunitinib sensitivity in resistant cells (Figure 8A–G). Treatment with the ULK1 agonist LYN‐1604 in PDZK1‐deficiency cells markedly enhanced their sunitinib sensitivity (Figure 8H). To validate the efficacy of combination therapy, we constructed a subcutaneous tumor model with low PDZK1 expression. We found that tumors with PDZK1 low expression were insensitive to sunitinib monotherapy. However, combination treatment with LYN‐1604 significantly reduced tumor weight and volume. This combination also enhanced autophagy‐related protein expression and reduced LD accumulation, demonstrating the anti‐tumor effects of lipophagy activation (Figure 8I–L).

Our study revealed a novel molecular mechanism of sunitinib resistance driven by LD accumulation. For the first time, we propose a combination therapy with LYN‐1604 and sunitinib to activate lipophagy, demonstrating synergistic anti‐tumor effects. This approach, distinct from conventional therapies targeting lipid metabolism, offers a novel clinical strategy to restore LD homeostasis and overcome resistance.

Our study has limitations. The cohort of ccRCC patients receiving sunitinib treatment was relatively small and based on retrospective analysis. Therefore, the role of ULK1 as a predictive biomarker requires further validation through multicenter clinical trials and meta‐analyses. Additionally, preclinical safety evaluations of LYN‐1604 are required, with particular focus on dose‐response relationships and long‐term toxicity.

In conclusion, our study unveiled a novel autophagy‐dependent pathway for LD degradation in ccRCC. PDZK1 inhibits tumor progression and sunitinib resistance by upregulating ULK1 and activating lipophagy. Preclinical studies suggest that the combination therapy with LYN‐1604 can improve the anti‐tumor effect of sunitinib by reducing LD accumulation. Furthermore, ULK1 could serve as a predictive biomarker of sunitinib efficacy, potentially enabling personalized treatment strategies for ccRCC patients.

## 4 Materials and Methods

### 4.1 Cell Lines and Cell Culture

The ccRCC cell lines 786‐O, 769‐P, Caki‐1, and ACHN were obtained from the National Infrastructure of Cell Line Resource (Beijing, China). 786‐O and 769‐P cells were cultured in RPMI‐1640 medium (Gibco, USA) supplemented with 10% fetal bovine serum (#SE100, Vistech, New Zealand) and 1% penicillin‐streptomycin solution (#P1400, Beijing Solarbio, China). ACHN cells were cultured in DMEM medium (Gibco, USA) with 10% fetal bovine serum and 1% penicillin‐streptomycin solution. All cell lines were maintained at 37°C in a humidified incubator with 5% CO_2_.

### 4.2 Antibodies and Reagents

Cis‐9‐Octadecenoic acid (#IC1350) was obtained from Solarbio (Beijing, China); 3‐Methyladenine (#S2767) and LYN‐1604 (#S8597) from Selleck (Houston, Texas, USA); Rapamycin (#HY‐10219), IWR‐1 (#HY‐12238), CHIR‐98014 (#HY‐13076), and Sunitinib (#HY‐10255A) from MedChemExpress (Shanghai, China).

Small interfering RNAs (siRNAs) were sourced from Sangon Biotech (Shanghai, China). PDZK1 shRNA and control shRNA were obtained from Santa Cruz Biotechnology (Dallas, Texas, USA). The Ad‐mCherry‐GFP‐LC3B plasmid (C3011) was purchased from Beyotime (Shanghai, China), and Lv‐PDZK1 was designed by GeneChem (Shanghai, China). Plasmids were supplied by Youbio Biological Technology (Changsha, China). siRNA and shRNA sequences are provided in Table S4.

Primary antibodies used are as follows: PDZK1 (#sc‐360964) and LAMP2 (#sc‐19991) from Santa Cruz Biotechnology (Dallas, Texas, USA). β‐Actin (#PTM‐5028), SQSTM1 (#PTM‐6434), and Lamin B (#PTM‐6357) from PTM BIO (Hangzhou, China). MAP1LC3B (#3868), ULK1 (#8054), and LEF1 (#2230) from CST. Importin α5 (#18137) from Proteintech (Wuhan, China).

### 4.3 Co‐Immunoprecipitation (Co‐IP)

Treated cells were lysed in non‐denaturing lysis buffer (#C1050, Applygen, China) with protease inhibitors. After centrifugation at 12 000 rpm for 15 min at 4°C, the supernatant was incubated with magnetic beads and antibodies at 4°C for 6 h. The beads were washed with 0.1% Tween‐20 buffer for three times, and the bound proteins were eluted by boiling in sample preparation buffer for Western blotting.

### 4.4 Western Blotting

Protein samples were separated by 8%–12% SDS‐PAGE and transferred onto PVDF membranes (Immobilon‐P; EMD Millipore). The membranes were blocked with 5% nonfat milk for 1 h, incubated with primary antibodies overnight at 4°C, and then with secondary antibodies for 1 h at room temperature. Protein signals were detected by ECL (#RPN2235, GE, USA) using an Amersham Imager 600 (GE, USA).

### 4.5 RNA Isolation and Quantitative Real‐Time PCR (RT‐qPCR)

Total RNA was extracted with RNA‐Quick Purification Kit (#RN001, esunbio, Shanghai) and reverse‐transcribed using the reverse transcription kit (#E047, Novoprotein, Suzhou).

RT‐qPCR was conducted using specific primers (Table S5) and SYBR Green master mix (#A25742, Thermo). Relative expression levels were calculated using the 2^−ΔΔCt^ method.

### 4.6 Immunohistochemistry (IHC)

Kidney and tumors samples were fixed in 4% paraformaldehyde (#P0099, Beyotime, Shanghai) and stained with the following antibodies: LEF1 (#2230, 1:100, CST, USA), MAP1LC3B (#3868, 1:100, CST, USA), ULK1 (#8054, 1:100, CST, USA), and PDZK1 (#360964, 1:50, Santa Cruz, USA).

Immunostaining was quantified using the histochemical score (H‐score) method, which integrates both staining intensity and the proportion of positive cells. Staining intensity was graded as 0 (negative, no staining), 1 (weak, light brown), 2 (moderate, brown), or 3 (strong, dark brown). The percentage of positive cells was recorded in 10% increments from 0% to 100%. The H‐score was calculated according to the formula H‐score = Σ(Pi × Ii), where Pi represents the percentage of cells at each intensity level, and Ii represents the intensity score, for clarity. Positive immunoreactivity was defined as nuclear staining for LEF1 and Ki67, and cytoplasmic staining for PDZK1, MAP1LC3B, and ULK1.

For each tissue section, an investigator blinded to experimental groups randomly selected five non‐overlapping high‐power fields (HPFs, ×200 magnification). Selection criteria included: (1) intact tissue architecture without artifacts, folding, or detachment; (2) absence of necrosis, hemorrhage, or inflammatory infiltrates; (3) adequate tumor cellularity with preserved nuclear morphology. All fields were photographed under standardized exposure settings. H‐scores were independently assessed by two board‐certified pathologists blinded to treatment allocation, and the mean value of thescores was used for statistical analysis. Data are presented as mean H‐scores from five fields per sample (n = 5 per group).

### 4.7 Immunofluorescence

Cells on coverslips were fixed with 4% paraformaldehyde, blocked with 3% BSA/PBS, and incubated with primary antibodies. Alexa Fluor 488 goat anti‐mouse (#A11001, Thermo Fisher, USA) or Alexa Fluor 594 donkey anti‐rabbit (#A21207, Thermo Fisher, USA) secondary antibodies were used for visualization. Nuclei were counterstained with DAPI (#P0131, Beyotime, Shanghai) and imaged using a LEICA SP8 confocal laser scanning microscope.

### 4.8 ORO Staining

Fixed RCC cells were rinsed with 60% isopropanol, stained with Oil Red O (ORO) stain kit (#G1262, Solarbio, Beijing), and counterstained with hematoxylin. Lipid droplet area and the number of lipid droplets per cell were quantified using Fiji (ImageJ).

### 4.9 BODIPY Staining

Cells were fixed with 4% formaldehyde, and stained with BODIPY 493/503 (#D3922, Thermo Fisher, USA) in the dark. Images were captured using a LEICA SP8 confocal laser scanning microscope and analyzed with Fiji (ImageJ).

### 4.10 Mouse Study

All animal experiments followed NIH guidelines and were approved by the Animal Use and Care Committee of Capital Medical University (approval numbers AEEI‐2020‐133 and AEEI‐2018‐065).

For subcutaneous xenograft models, 786‐O cells (5 × 10^6^) suspended in 100 µL PBS were injected into the left flank of six‐week‐old BALB/c nude mice. Mice were housed under controlled conditions (22 ± 1°C, 50 ± 1% humidity, 12/12‐h circadian rhythm). Both male and female mice were used, with consistent results observed across sexes.

Tumor growth was measured every five days, and tumor volume was calculated using the formula (Length × Width^2^) / 2. Euthanized was performed at endpoint for tumor collection.

### 4.11 Statistical Analysis

Data are presented as mean ± standard deviation (SD) from at least three independent experiments. Student's t‐test was used for two‐group comparisons, while one‐way ANOVA with Tukey's test was applied for three or more groups. Pearson correlation was used for Gaussian‐distributed variables, and Spearman correlation for non‐Gaussian data. Survival analysis was performed using the Kaplan–Meier method with a log‐rank test. Statistical significance was set at p < 0.05 (two‐tailed). Significance levels: ns, not significant; ^*^ *p* < 0.05; ^**^ *p* < 0.01; ^***^ *p* < 0.001. All analyses were conducted using GraphPad Prism v9.0 (GraphPad Software, San Diego, USA). A p‐value < 0.05 was taken as statistically significant.

### 4.12 Protein‐Protein Docking

The structures of PDZK1 (UniProt ID: Q5T2W1) and LEF1 (UniProt ID: Q9UJU2) were retrieved from the AlphaFold Database (https://www.alphafold.ebi.ac.uk). PDZK1 was set as the receptor and LEF1 as the ligand for interaction prediction using HDOCK software with default parameters [35]. Structural were visualized in PyMOL (http://pymol.sourceforge.net/).

## Author Contributions

**Junqi He**: investigation, funding acquisition, project administration, supervision. **Xuan Qi**: conceptualization, writing – original draft, validation, visualization. **Yu Guo**: writing – review and editing, validation, visualization. **Xiaomei Yang and Ran Song**: validation. **Haibo Wang and Qiong Qin**: methodology. **Yumeng Yang and Haixing Zhou**: formal analysis. **Meihan Hu and Yan Zhang**: visualization. **Duiping Feng**: funding acquisition.

## Funding

This work was supported by the National Natural Science Foundation of the People's Republic of China (Grants Nos. 82273965 and 81972732), the Beijing Municipal Natural Science Foundation (Nos. 7242009 and 7222006), and the Natural Science Foundation of Shanxi Province (Grant No. 20210302123258).

## Ethics Approval and Consent to Participate

The research involving ccRCC samples was approved by the Ethics Committee of Capital Medical University (2017SY09) and the First Hospital of Shanxi Medical University (2022HLL001). All participants agreed to participate in the study and signed the informed consent document.

All animal experiments were approved by the Animal Use and Care Committee of Capital Medical University (AEEI‐2020‐133; AEEI‐2018‐16). All of the animal experiments were performed by the relevant guidelines and regulations and were approved by the Animal Use and Care Committee of Capital Medical University.

## Conflicts of Interest

The authors declare no conflicts of interest.

## Supporting information




**Supporting File 1**: advs74385‐sup‐0001‐SuppMat.docx.

**Supporting File 2**: advs74385‐sup‐0002‐TableS1‐S5.xlsx.

**Supporting File 3**: advs74385‐sup‐0003‐Data.docx.

## Data Availability

The data that support the findings of this study are available from the corresponding author upon reasonable request.
