# Supplementary material for: PDZK1‐ULK1 Axis Triggers Lipophagy to Inhibit Tumor Progression and Sunitinib Resistance in Clear Cell Renal Cell Carcinoma
Source: Adv Sci (Weinh). 2026 Feb 16;13(23):e11606. doi: 10.1002/advs.202511606 (PMC13104086; doi:10.1002/advs.202511606)
Supplement: Supplementary file 1 — Supporting File 1: advs74385‐sup‐0001‐SuppMat.docx. [file ADVS-13-e11606-s003.docx]

Supporting Information

**PDZK1-ULK1 Axis Triggers Lipophagy to Inhibit Tumor Progression and Sunitinib Resistance in Clear Cell Renal Cell Carcinoma**

Xuan Qi, Yu Guo, Yumeng Yang, Haibo Wang, Xiaomei Yang, Ran Song, Qiong Qin ^1^, Yan Zhang, Meihan Hu, Haixing Zhou, Duiping Feng, Junqi He^*^

**Supplementary Figures**

**Figure S1.**

**
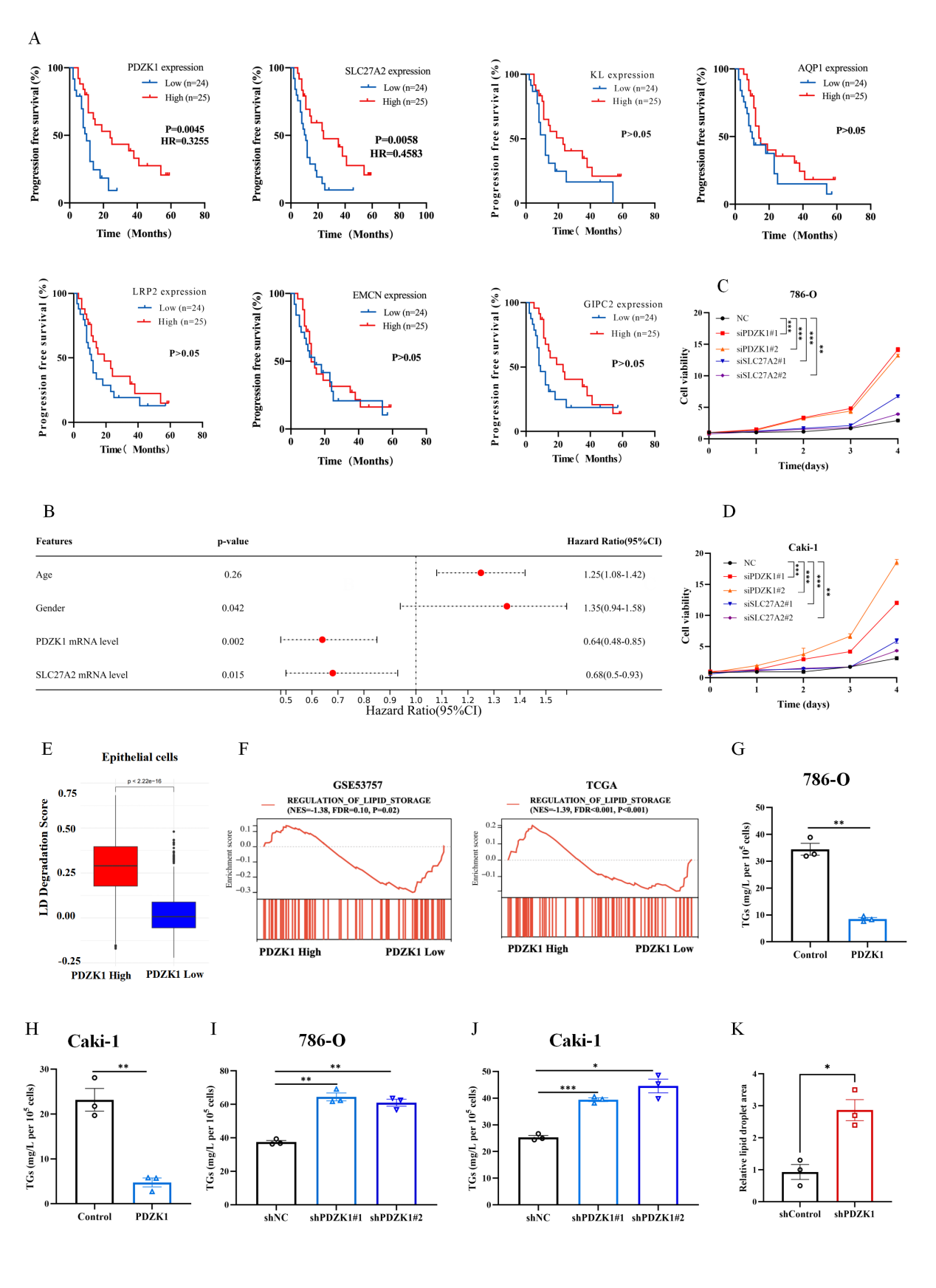
**

**Figure S1. PDZK1 inhibits lipid accumulation and correlates with favorable prognosis in ccRCC.**

1. Kaplan-Meier survival analysis of seven candidate genes in the E-TAB-3267 dataset. Patients were stratified into high- and low-expression groups based on the mRNA expression level of each candidate. Survival differences were determined by the log-rank test. P-values are indicated in each panel.
2. Cox multivariate analysis indicated that PDZK1 mRNA level and SLC27A2 mRNA level were independent factors for prognosis prediction of ccRCC patients.

**C-D.** Both PDZK1 and SLC27A2 knockdown significantly increased ccRCC cell proliferation. Thel proliferation of 786-O (B) or Caki-1 (C) cells transfected with siPDZK1, siSLC27A2 or pcDNA3.0 were assessed by CCK-8.

**E.** Box plots showing LD degradation score within epithelial cell subpopulation based on PDZK1 expression. Statistics are accessed with two-sided Wilcoxon rank sum test.

**F.** GSEA enrichment plots showing significant enrichment of lipid storage in ccRCC specimens with low PDZK1 expression from GSE53757 and TCGA dataset.

**G-J.** Triglyceride (TG) levels were measured in PDZK1 knockdown or PDZK1 overexpressing 786-O and Caki-1 cells. Cells transfected with vector or shPDZK1 (F, G). Cells transfected with vector or PDZK1 overexpression (H, I). Data are presented as mean ±SD. from three technical replicates. **p < 0.01, ***p < 0.001.

**K.** Quantification of lipid droplets in orthotopic tumor tissues using ImageJ (J). Data are presented as mean ±SD (n=3 replicates). *p < 0.05.

**Figure S2.**

**
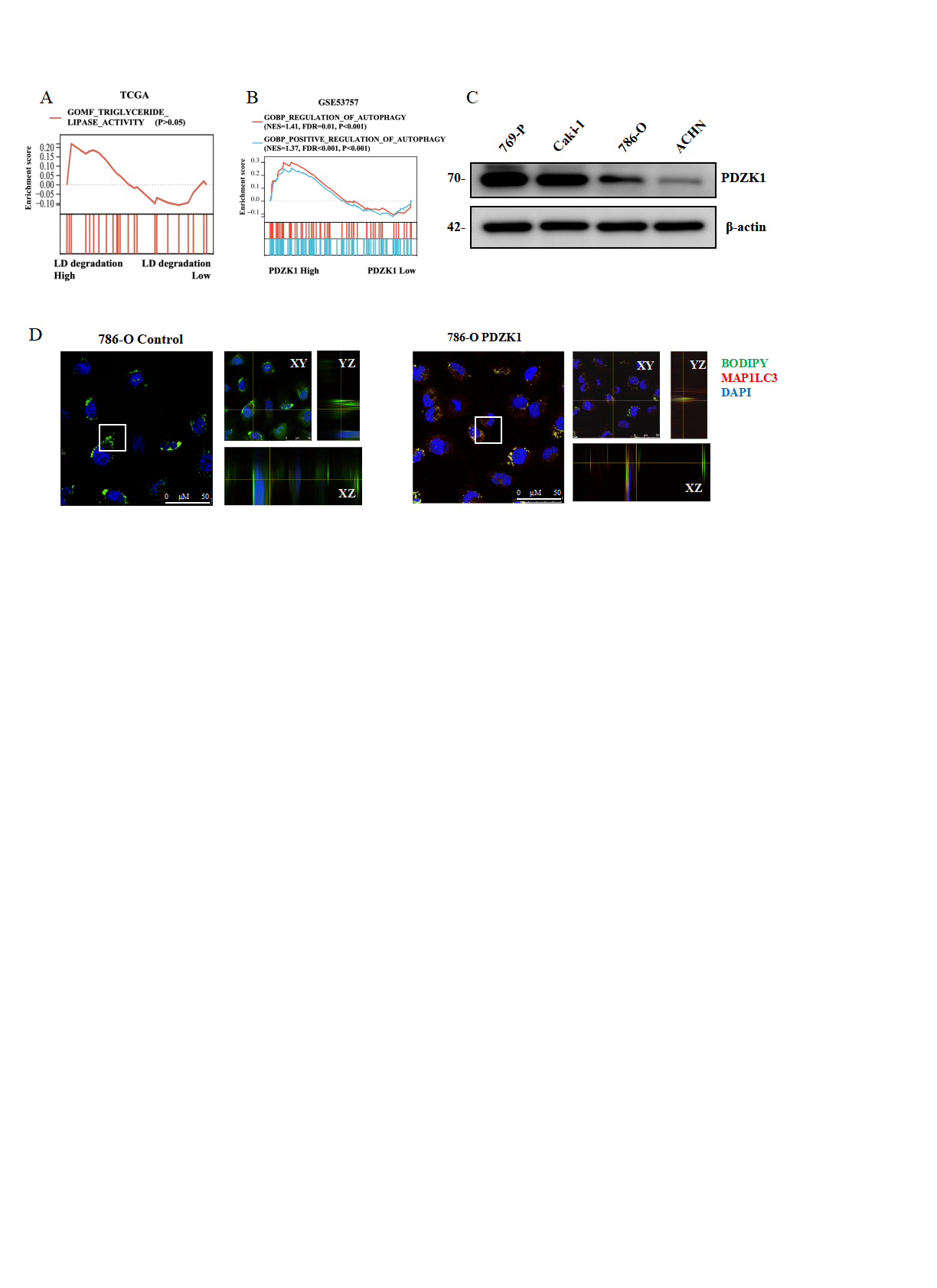
**

**Figure S2. PDZK1 activates autophagy in ccRCC.**

1. GSEA enrichment plots indicating no significant enrichment of the lipolysis pathway in ccRCC specimens from TCGA dataset. Patients were stratified into low and high LD degradation score groups.
2. GSEA enrichment plots demonstrating significant enrichment of autophagy-related pathway in ccRCC specimens with high PDZK1 expression from GSE53757 dataset.
3. Western blot analysis showing baseline PDZK1 expression in 769-P, Caki-1, 786-O and ACHN cell lines.
4. Colocalization analysis of BODIPY (green) and MAP1LC3B (red) signals across sequential z-axis layers acquired by confocal microscopy. The x-axis indicates the z-axis position (depth), and the y-axis represents signal intensity or colocalization coefficients.

**Figure S3.**

**
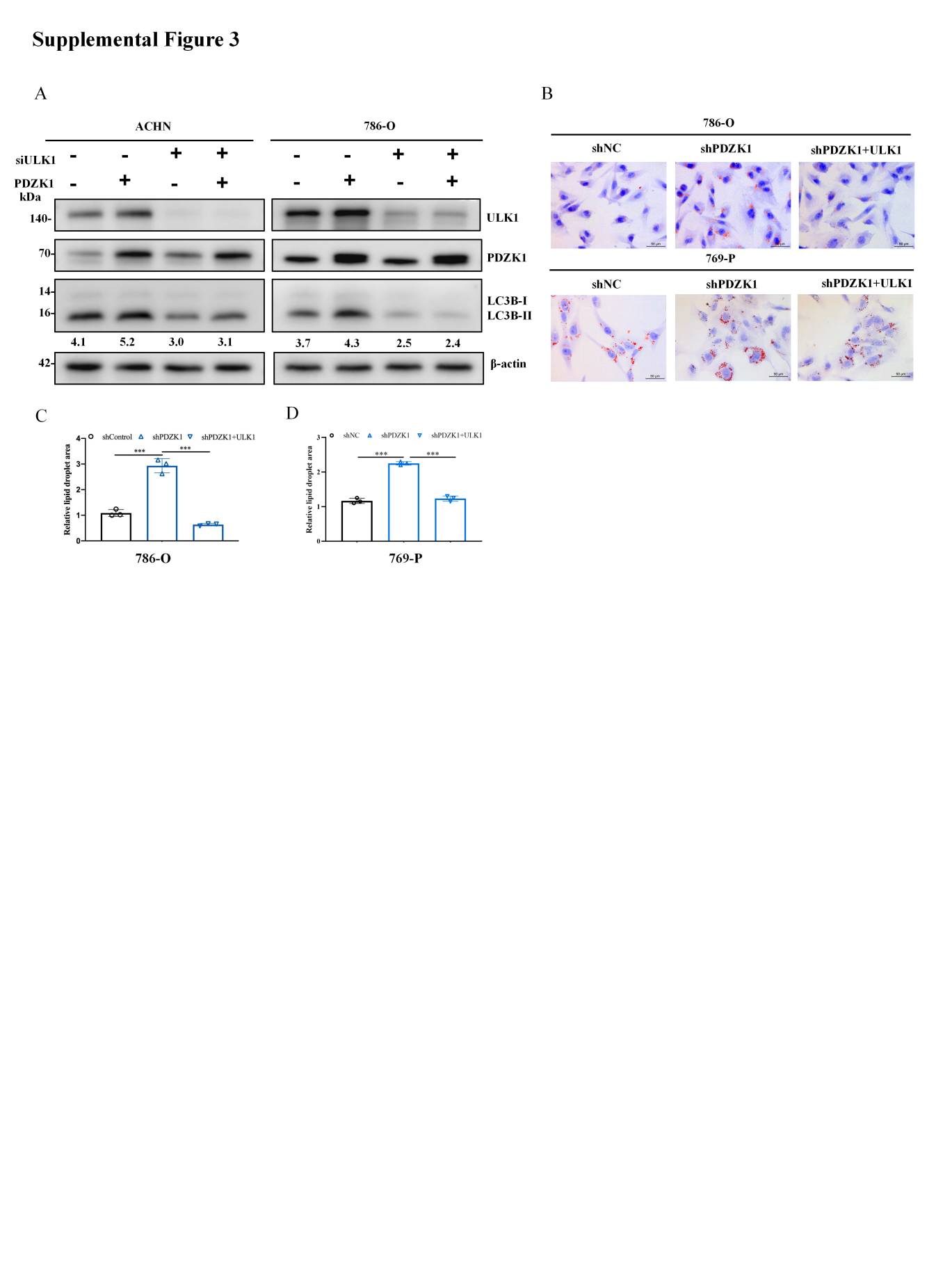
**

**Figure S3. PDZK1 activates lipophagy by upregulating ULK1 expression.**

**A.** Knockdown of ULK1 reversed PDZK1 overexpression-induced autophagy activation, as shown by Western blot analysis.

**B-D**. Overexpression of ULK1 reversed PDZK1 knockdown-induced lipid storage, as shown by Oil Red O staining (B, C). Scale bar, 50 μm. Lipid droplet quantification using ImageJ (D). Data are presented as mean ±SD. from three technical replicates. ***p < 0.001.

**Figure S4.**

**
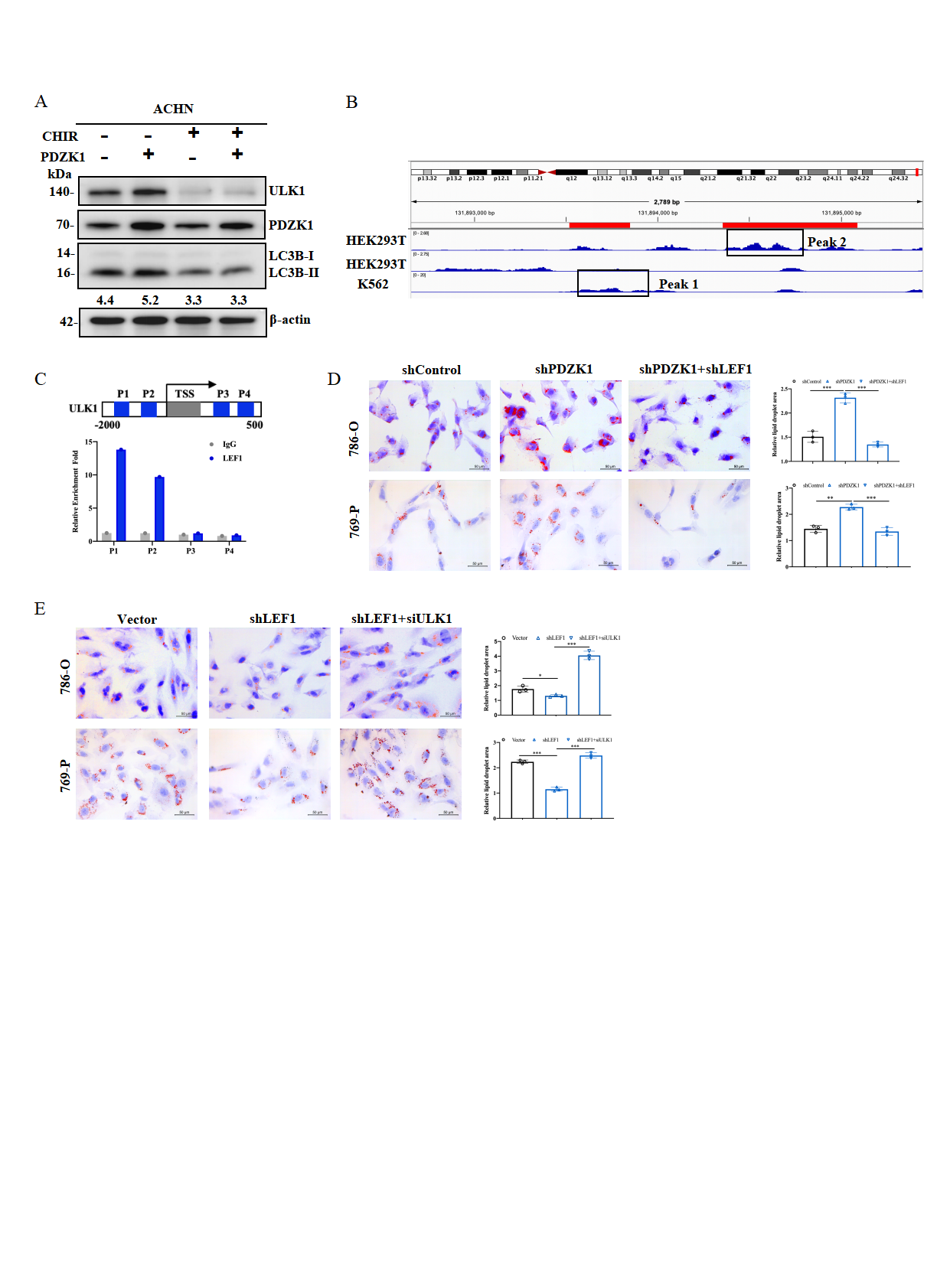
**

**Figure S4. LEF1 mediates the transcription regulation of ULK1 in PDZK1-dependent manner.**

1. Activation of the Wnt/β-catenin pathway decreases ULK1 protein levels, reversing the elevation caused by PDZK1 overexpressing in ACHN cells.
2. The LEF1 binding peak on the ULK1 genome in HEK293T and K562 cells were examined from GSE105382 and GSE91682 dataset.
3. The CUT&Tag-qPCR analysis of LEF1.
4. To knockdown LEF1 expression in 786-O cells, both with and without PDZK1 knockdown, was established through Oil Red O staining. Data are presented as mean ±SD from three technical replicates.
5. To knockdown ULK1 expression in 786-O cells, both with and without LEF1 knockdown, was established through Oil Red O staining. Quantification of lipid droplet with ImageJ. Data are presented as mean ±SD. from three technical replicates.

In all statistical plots, data are shown as mean ± SD, one-way ANOVA (Figure S4D,E) was used to determine statistical signiﬁcance (*  < 0.05, **p < 0.01, ***p < 0.001).

**Figure S5.**

**
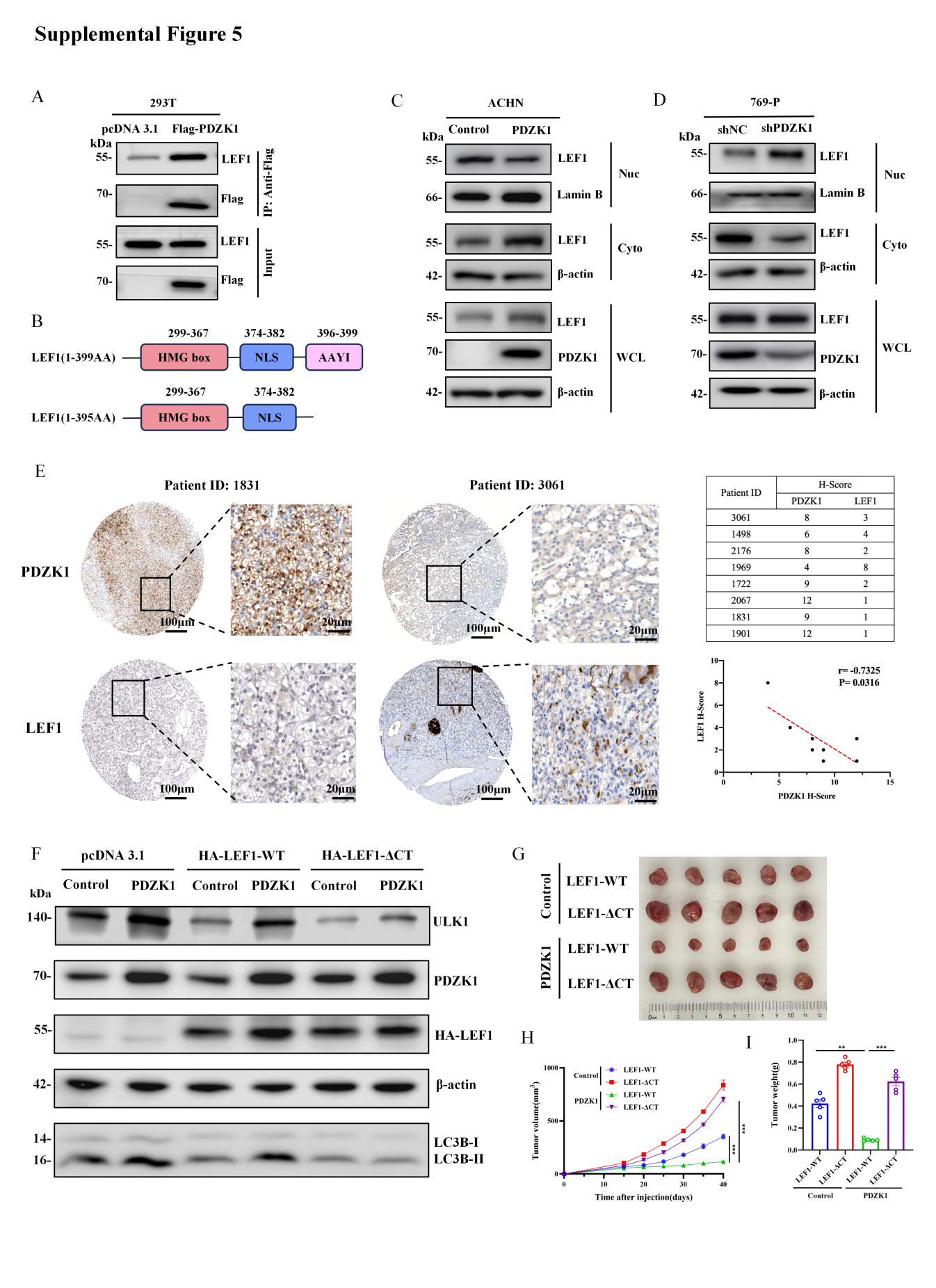
**

**Figure S5.** **PDZK1 interacts with LEF1 to restrict its nuclear translocation.**

1. Co-IP assay confirming the association between PDZK1 and LEF1 in HEK293T cells.
2. Schematic diagrams of full-length LEF1 and the LEF1 C-terminal truncation mutant (LEF1-ΔCT).
3. Western blot analysis of nuclear and cytosolic fractions from control and PDZK1 overexpressing ACHN cells.

**D.** Western blot analysis of nuclear and cytosolic fractions from control and PDZK1 knockdown 769-P cells.

**E.** Immunohistochemical staining of LEF1 in ccRCC patients with low or high PDZK1 expression. Correlation analysis between PDZK1 protein level and nuclear LEF1 expression is shown.

**F.** Western blot analysis of ULK1 and LC3B expression in 786-O cells expressing empty vector or PDZK1, together with HA‑tagged LEF1-WT or LEF1-ΔCT (C‑terminally truncated).

**G-I.** Subcutaneous tumor growth in nude mice injected with 786-O cells expressing LEF1‑WT or LEF1‑ΔCT, together with control or PDZK1‑targeting shRNA (G). Tumor volume was monitored regularly (H), and final tumor weights are shown (I). Data represent mean ± SD (n=5); two-tailed, unpaired t-test. **p < 0.01, ***p < 0.001.

**Figure S6.**


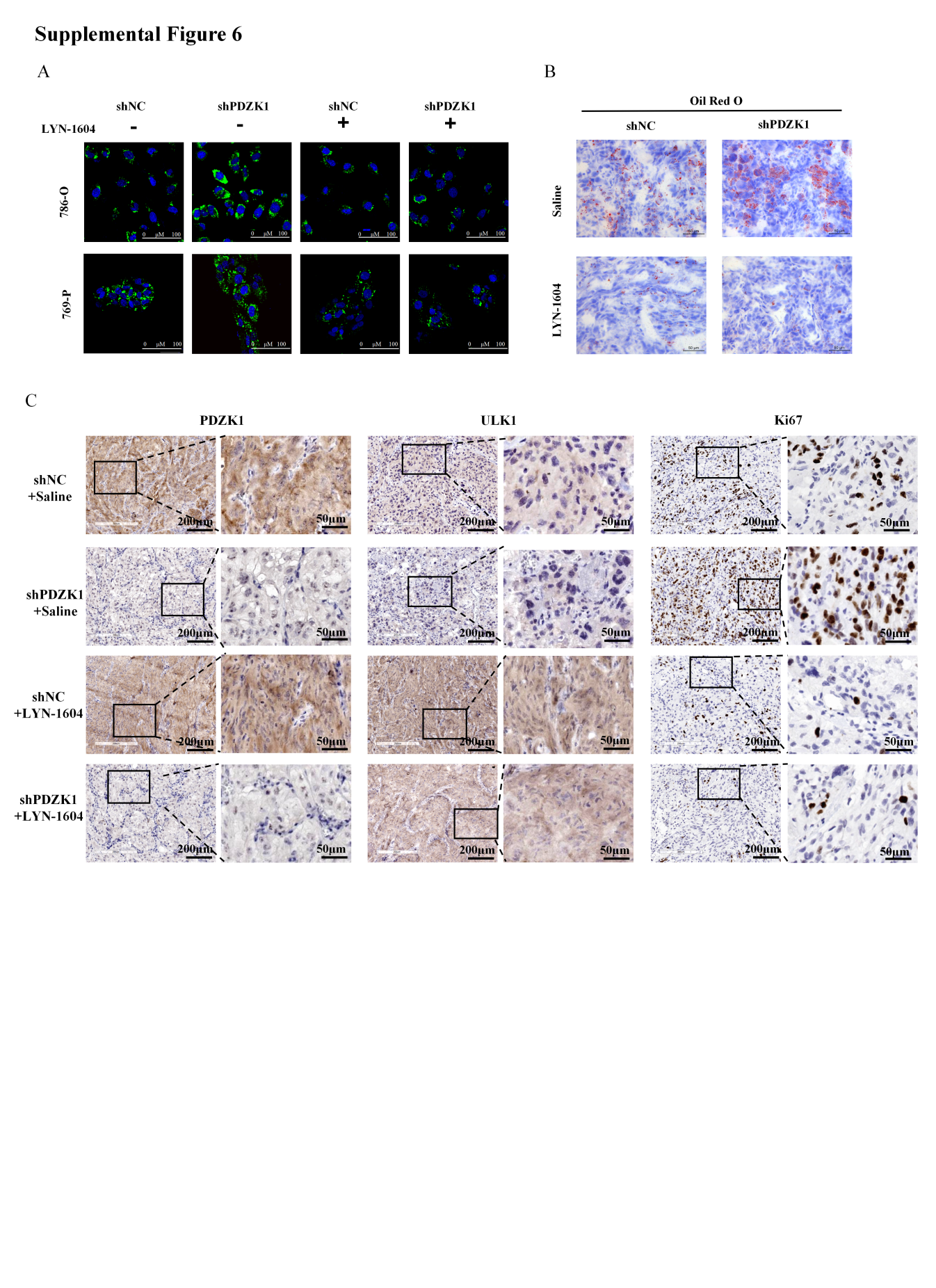


**Figure S6. The PDZK1-ULK1 axis inhibits tumor progression by activating lipophagy.**

1. Control and PDZK1 knockdown 786-O cells were treated with or without LYN-1604 for 24 h. Lipid droplets (green) were visualized by immunofluorescence assay and quantified using Image J. Scale bar, 100 μm.
2. Photomicrographs of Oil red staining in isolated tumor xenografts of the shControl + Saline, shPDZK1 + Saline, shControl + LYN-1604, and shPDZK1 + LYN-1604.
3. IHC staining for PDZK1, ULK1 and Ki67 in the isolated tumor xenografts of the shControl + Saline, shPDZK1 + Saline, shControl + LYN-1604, and shPDZK1 + LYN-1604.

**Figure S7.**

**
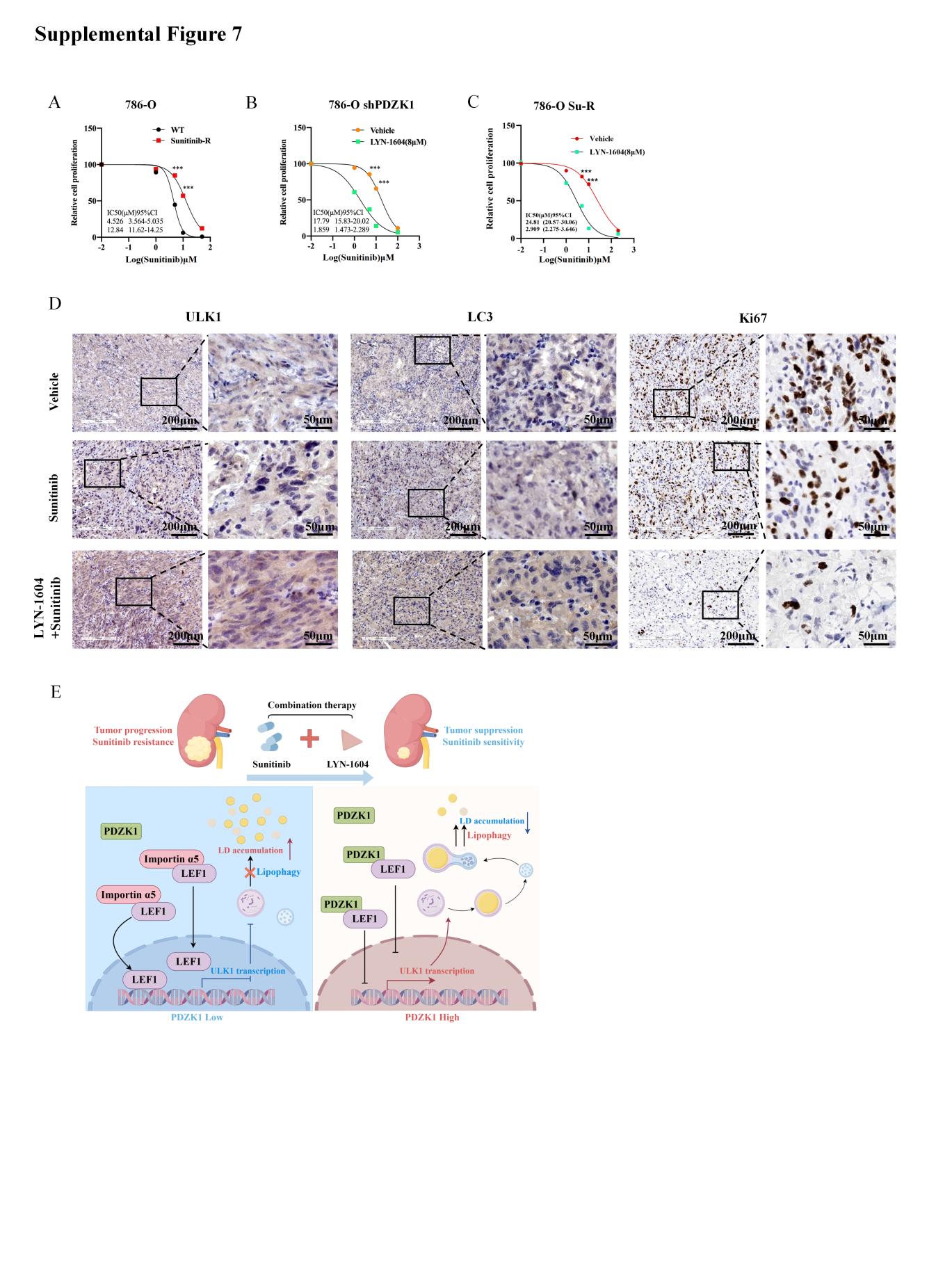
**

**Figure S7. ULK1 agonist sensitizes tumors with PDZK1 deficiency-induced high LDs accumulation to sunitinib.**

**A.** Establishment of sunitinib-resistant 786-O cells. Dose–response survival curves of sunitinib sensitive or sunitinib resistant 786-O cells exposed to increasing concentrations of sunitinib for 48 hours. Cell viability was assessed using the CCK-8 assay.

**B.** Viability of PDZK1 knockdown 786-O cells treated with vehicle or LYN-1604, followed by exposure to different concentrations of sunitinib and CCK8 assay.

**C.** Viability of sunitinib-resistant 786-O cells treated with vehicle or LYN-1604, followed by exposure to different concentrations of sunitinib and CCK-8 assay.

**D.** Xenograft assay of PDZK1 knockdown 786-O cells. The mice were treated with vehicle, sunitinib (20 mg/kg, daily gavage, 21 days), or LYN-1604 (50 mg/ kg, daily gavage, 21 days) combined with sunitinib (the condition as above). Representative images of subcutaneous tumors.

**E.** IHC staining for PDZK1, ULK1 and Ki67 in tumor xenografts from the shPDZK1 + vehicle, shPDZK1 + LYN-1604, and shPDZK1 + LYN-1604+ sunitinib groups.

**F.** Schematic summary of this study.

In all statistical plots, data are shown as mean ± SD (n=3 replicates), Two-tailed unpaired Student’s t test (Figure S7A-C) were used to determine statistical significance (***p < 0.001).
