# Supplementary material for: PDZK1‐ULK1 Axis Triggers Lipophagy to Inhibit Tumor Progression and Sunitinib Resistance in Clear Cell Renal Cell Carcinoma
Source: Adv Sci (Weinh). 2026 Feb 16;13(23):e11606. doi: 10.1002/advs.202511606 (PMC13104086; doi:10.1002/advs.202511606)

Full unedited blot for Figure 2C

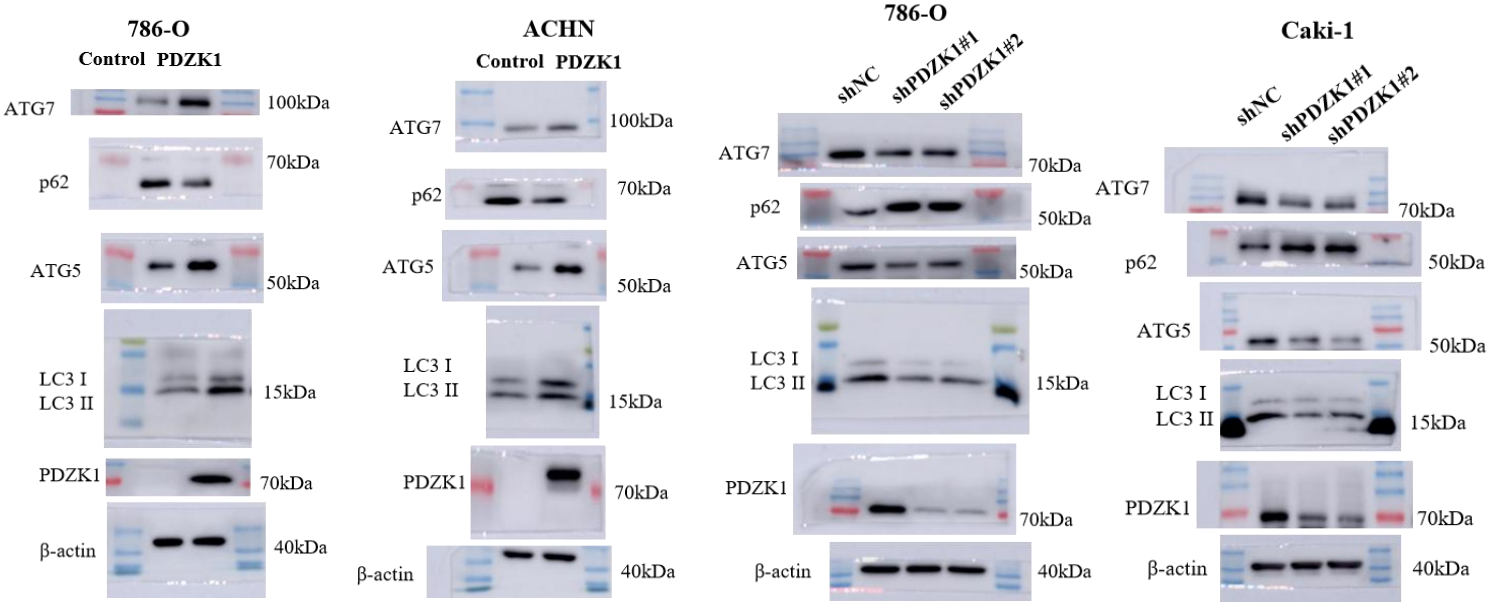

Full unedited blot for Figure 2D

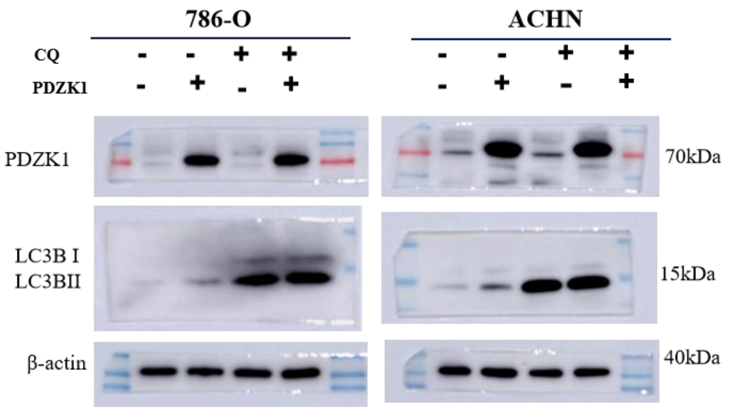

Full unedited blot for Figure 2E

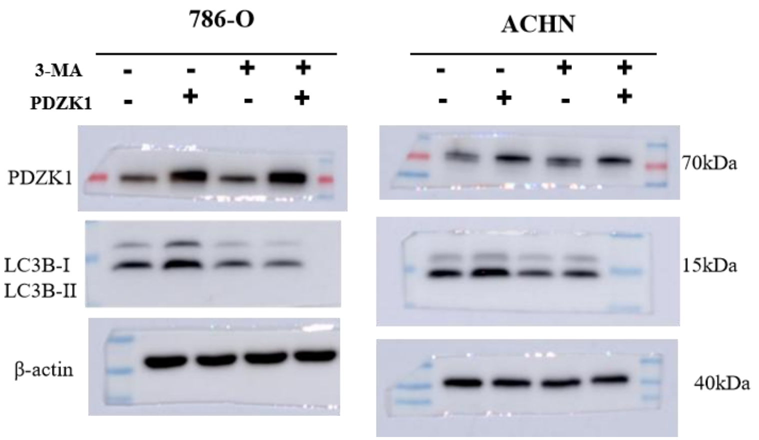

Full unedited blot for Figure 4F

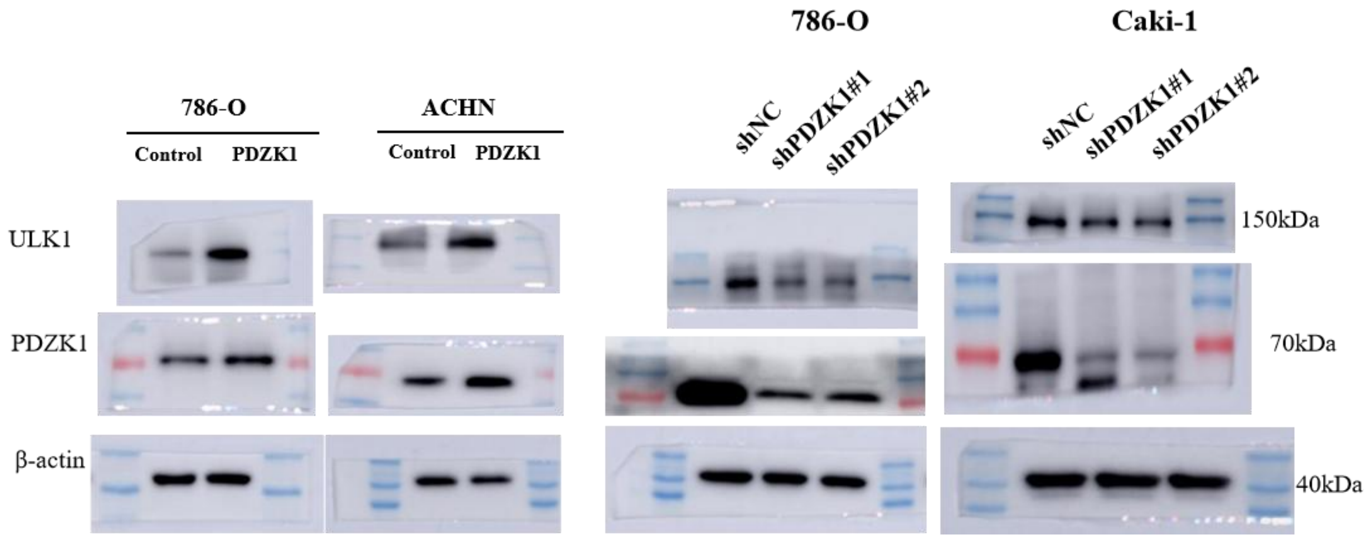

Full unedited blot for Figure 4H

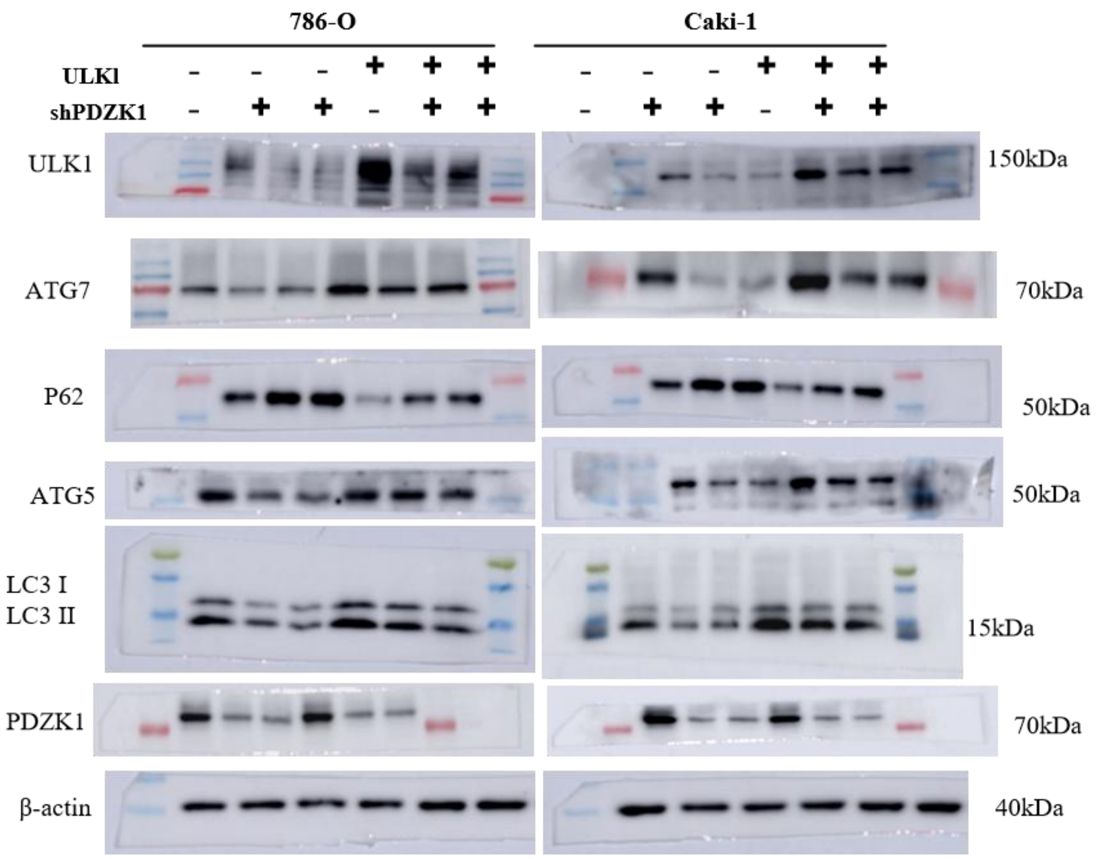

Full unedited blot for Figure 5F

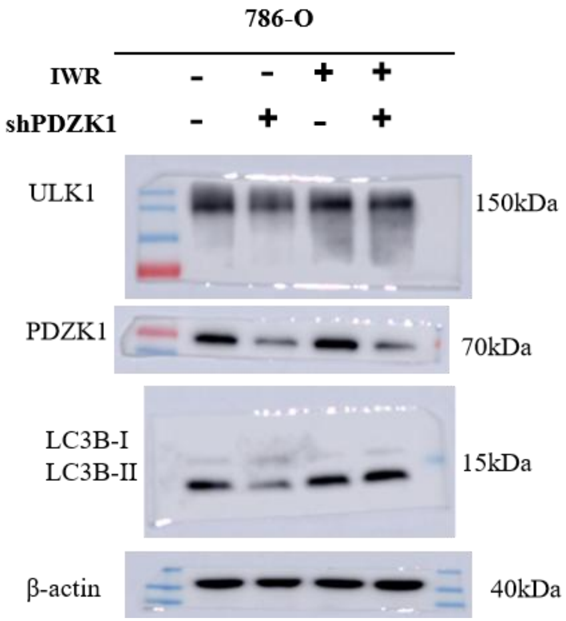

Full unedited blot for Figure 5G

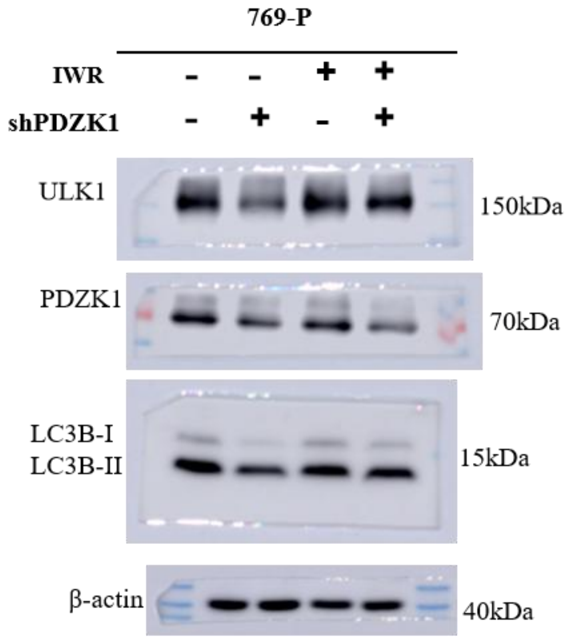

Full unedited blot for Figure 5H

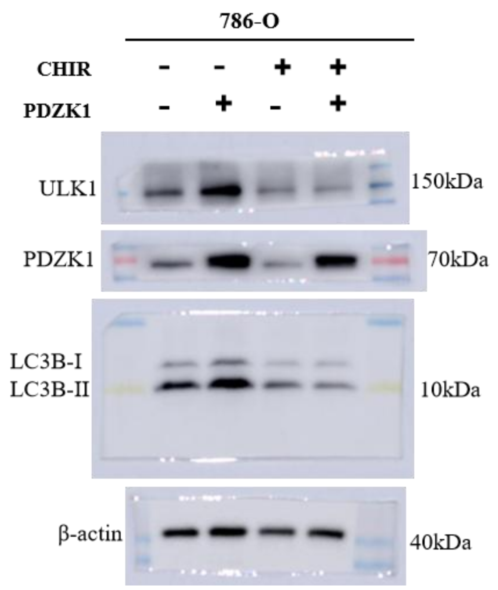

Full unedited blot for Figure 5P

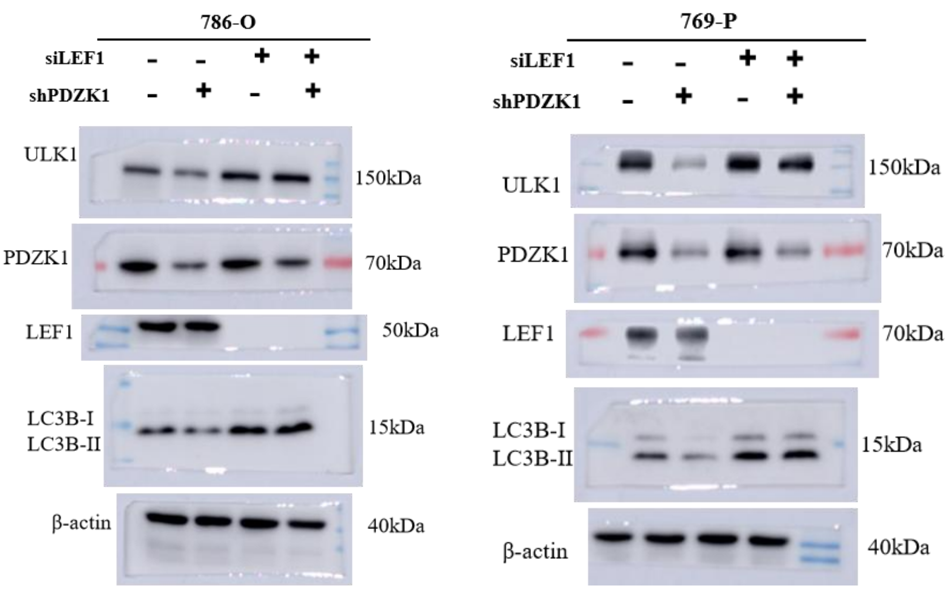

Full unedited blot for Figure 6A

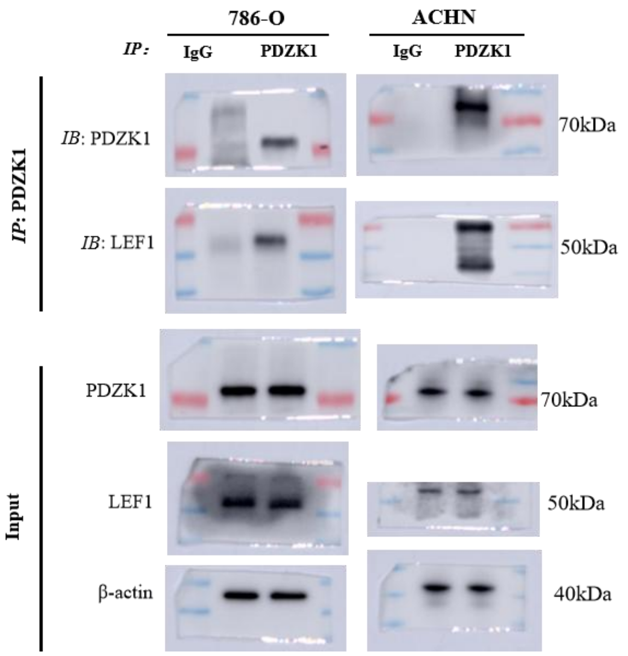

Full unedited blot for Figure 6B

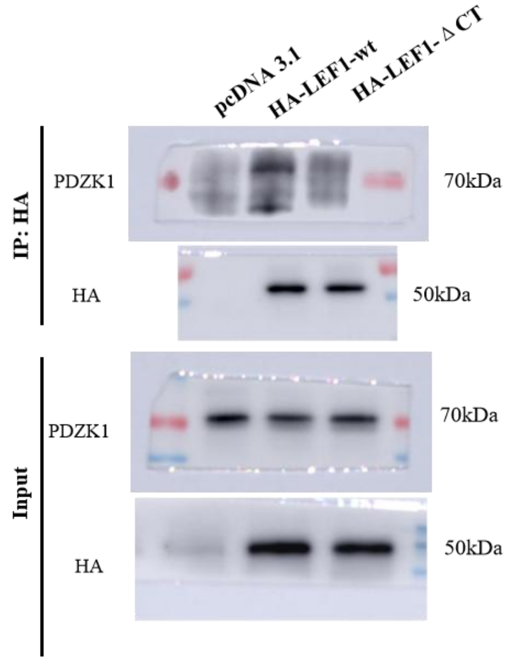

Full unedited blot for Figure 6D

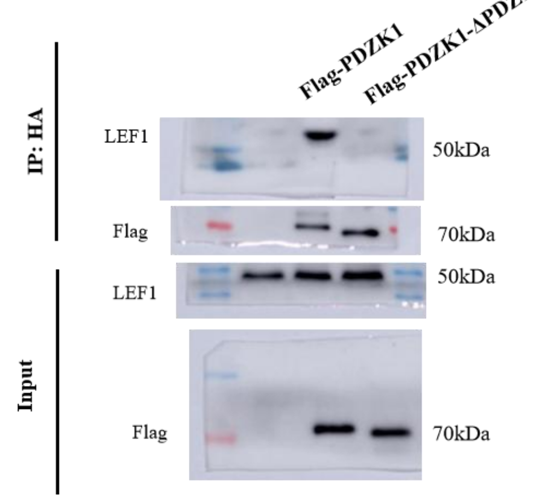

Full unedited blot for

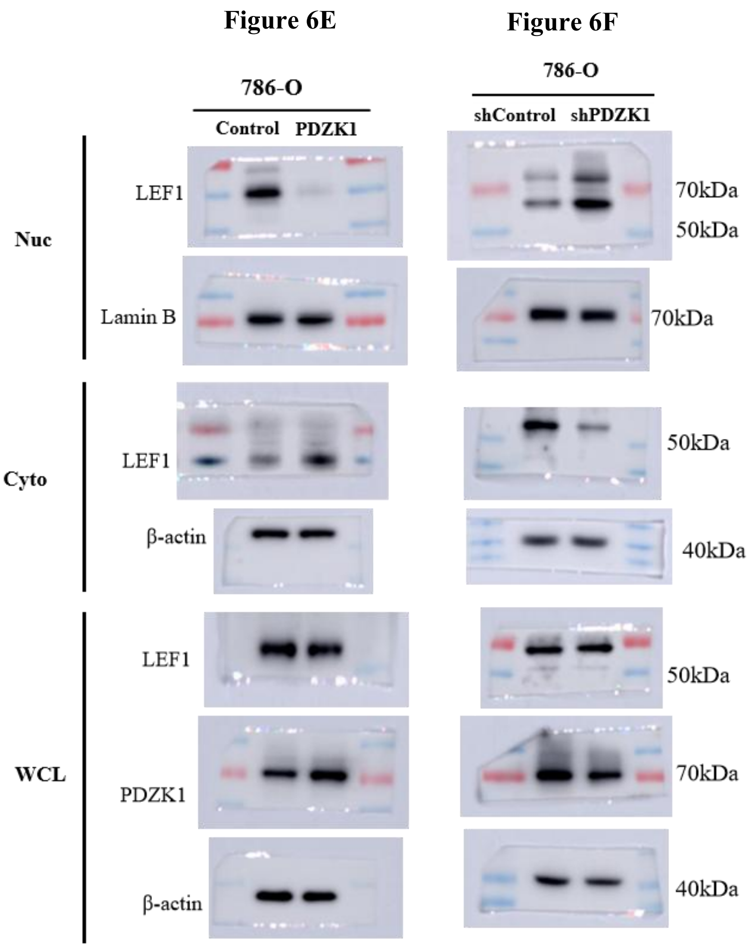

Full unedited blot for Figur 6H

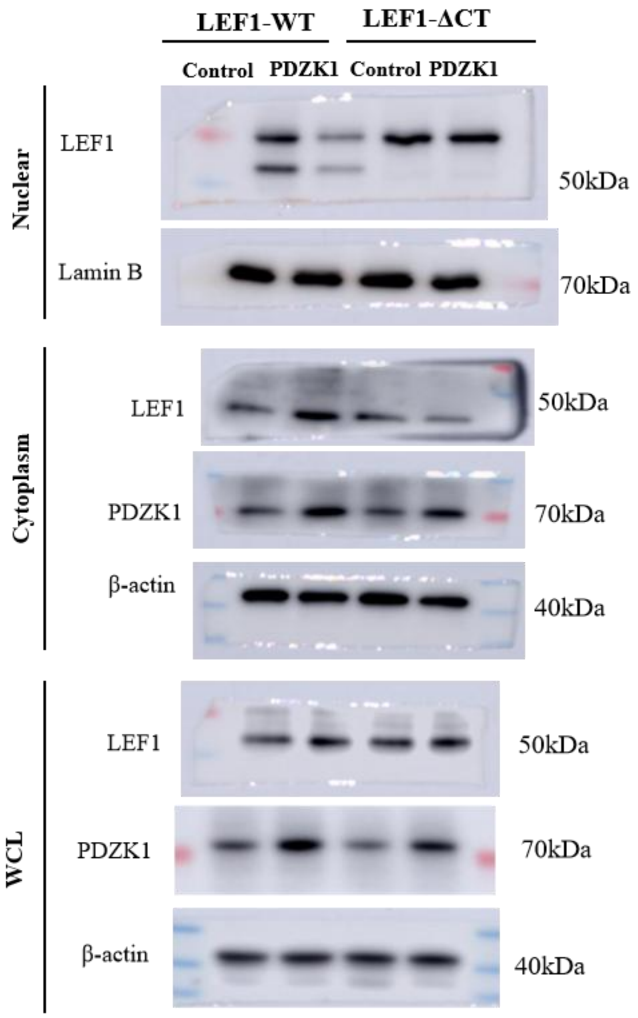

Full unedited blot for Figur 6L,M

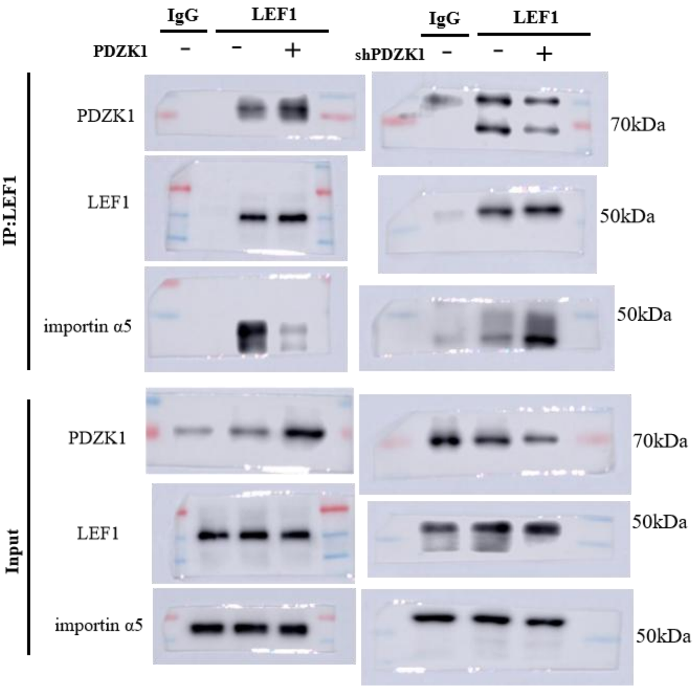

Full unedited blot for Figur 7E

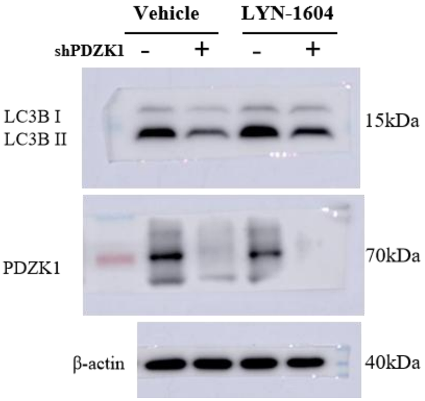

Full unedited blot for Figur 8G

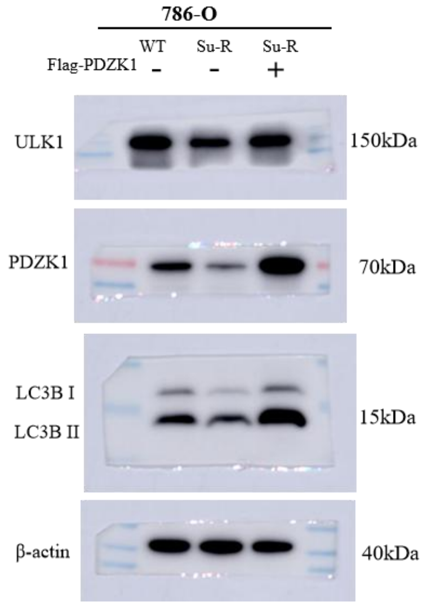

Full unedited blot for Supplemental Figure 2C

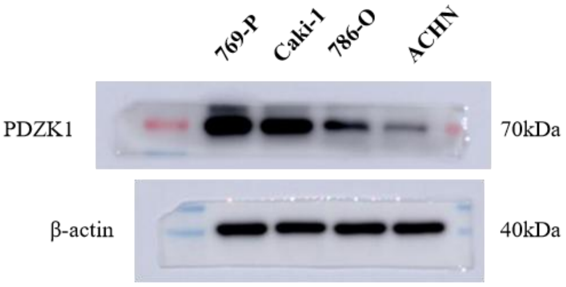

Full unedited blot for Supplemental Figure 3A

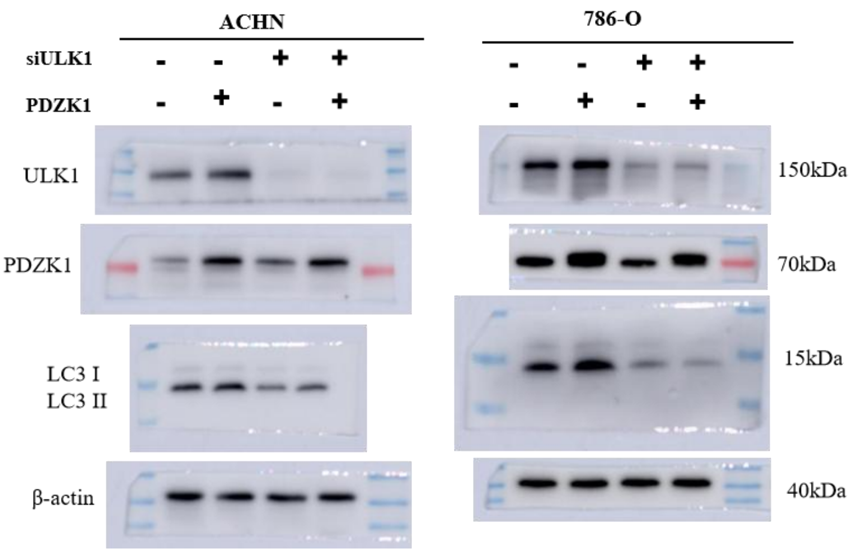

Full unedited blot for Supplemental Figure4A

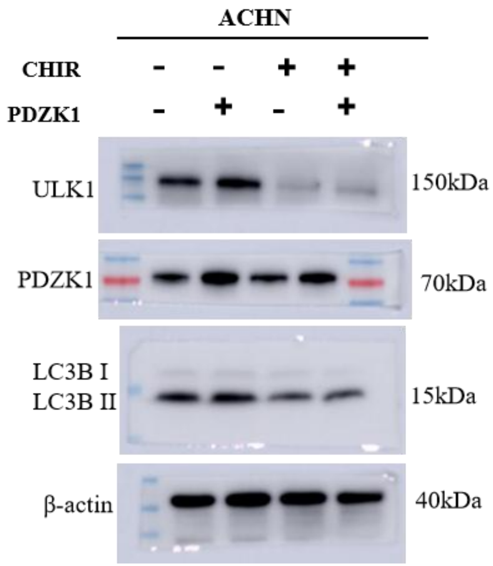

Full unedited blot for Supplemental Figure 5A

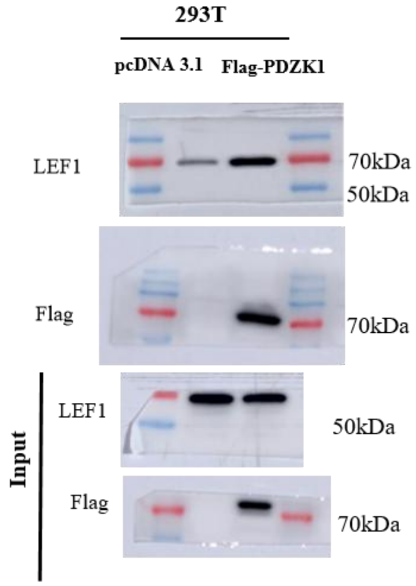

Full unedited blot for Supplemental Figure 5C,D

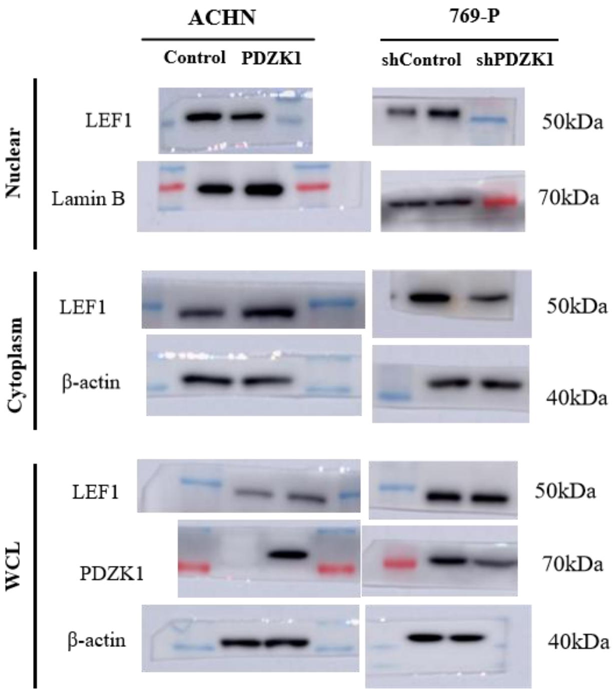

Full unedited blot for Supplemental Figure 5F

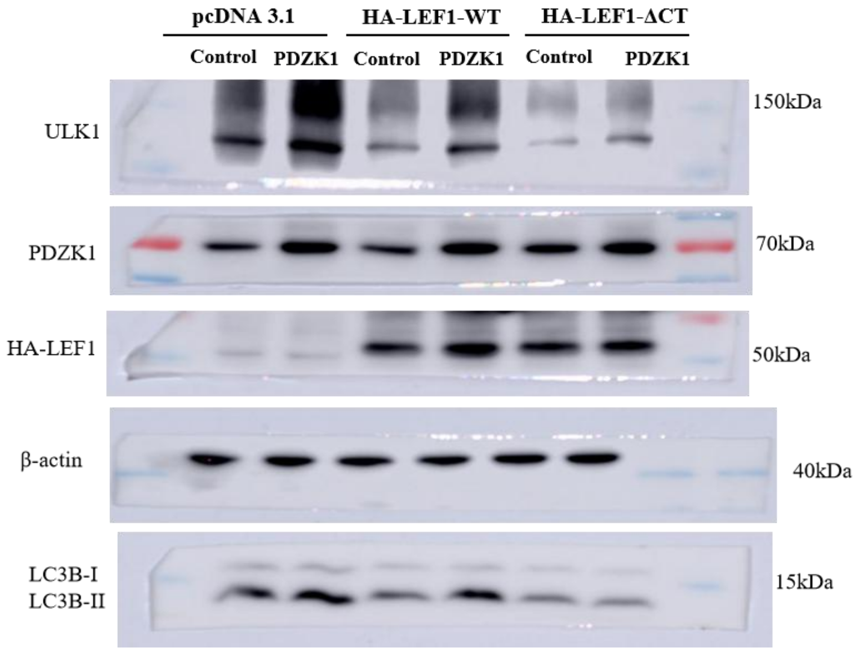

Supplement: Supplementary file 3 — Supporting File 3: advs74385‐sup‐0003‐Data.docx. [file ADVS-13-e11606-s001.zip › advs74385-sup-0003-Data/Original western blot.pdf]
